# Revealing the recent demographic history of Europe via haplotype sharing in the UK Biobank

**DOI:** 10.1073/pnas.2119281119

**Published:** 2022-06-13

**Authors:** Edmund Gilbert, Ashwini Shanmugam, Gianpiero L. Cavalleri

**Affiliations:** ^a^School of Pharmacy and Biomolecular Sciences, Royal College of Surgeons in Ireland, Dublin, D02 YN77, Ireland;; ^b^FutureNeuro SFI Research Centre, Royal College of Surgeons in Ireland, Dublin, D02 YN77, Ireland;; ^c^SFI Centre for Research Training in Genomics Data Science, School of Mathematics, Statistics & Applied Mathematics, National University of Ireland, Galway, H91 TK33, Ireland

**Keywords:** population genetics, demographic history, haplotypes, identity by descent

## Abstract

Haplotype-based analyses have recently been leveraged to interrogate the fine-scale structure in specific geographic regions, notably in Europe, although an equivalent haplotype-based understanding across the whole of Europe with these tools is lacking. Furthermore, study of identity-by-descent (IBD) sharing in a large sample of haplotypes across Europe would allow a direct comparison between different demographic histories of different regions. The UK Biobank (UKBB) is a population-scale dataset of genotype and phenotype data collected from the United Kingdom, with established sampling of worldwide ancestries. The exact content of these non-UK ancestries is largely uncharacterized, where study could highlight valuable intracontinental ancestry references with deep phenotyping within the UKBB. In this context, we sought to investigate the sample of European ancestry captured in the UKBB. We studied the haplotypes of 5,500 UKBB individuals with a European birthplace; investigated the population structure and demographic history in Europe, showing in parallel the variety of footprints of demographic history in different genetic regions around Europe; and expand knowledge of the genetic landscape of the east and southeast of Europe. Providing an updated map of European genetics, we leverage IBD-segment sharing to explore the extent of population isolation and size across the continent. In addition to building and expanding upon previous knowledge in Europe, our results show the UKBB as a source of diverse ancestries beyond Britain. These worldwide ancestries sampled in the UKBB may complement and inform researchers interested in specific communities or regions not limited to Britain.

## Significance

Recent haplotype sharing analyses in specific European populations have revealed fine-scale genetic differentiation that echoes history. An equivalent understanding across the whole European continent would place these insights into a wider context and extend understanding to underdescribed regions. Here, we leverage haplotype data from 5,500 European-ancestry individuals from the UK Biobank (UKBB) in a methodological approach to update and expand the European genetic landscape, extending coverage to regions in the southeast of Europe, identifying communities of high haplotype sharing that may be of interest to genetic mapping, such as Malta. Together, our results highlight European communities with diverse ancestries sampled within the UKBB and demonstrate the potential for insights to be made with other non-European ancestry communities using this dataset.

Recent studies utilizing haplotype- (or “chunk”-) based approaches (1) have demonstrated a wide-spread fine-scale genetic structure in multiple regional populations, particularly within Europe (2–9). These studies have revealed a varied genetic structure whose landscapes often echo historical groups or events, commonly utilizing individuals with genealogical ancestry from specific subregions and thus being able to compare geographic and genetic similarities. This work has understandably focused on specific regions, elucidating their fine-grained histories and genetic structure, which is fundamental in designing studies of rare variation and its association with traits of interest in specific populations. However, these geographically localized studies have meant that a similar understanding of the haplotypic landscape across the span of Europe has yet to be attained.

At the same time as improvements into the study of genetic history with haplotype-based coancestry information (1, 10, 11), identical-by-descent (IBD) segments have been increasingly used in population history inference (12, 13). IBD segments are contiguous spans, haplotypes, of the genome that are identical between two individuals. This state of shared identity reflects at that the haplotypes are copies that descend from a common ancestor in the pairs’ “recent” genealogical past. Patterns of IBD segment sharing between groups of individuals from the same population reflect that population’s demographic history, including population structure (12, 13), recent effective population size (14, 15), and migration rates (5, 16). Furthermore, IBD segments can also be used to identify genomic regions carrying disease-related variants (17–19)—highlighting the wide applicability of haplotype data in understanding history and disease. Self-matching of IBD segments, so-called “runs of homozygosity” (ROH), have additionally been used to both explore population history (20, 21) and disease association (22)—especially in small and/or endogamous populations where ROH are more common than in large outbred populations.

Motivated by recent haplotype-based analyses of specific European populations (2–4, 7, 23) and developments in haplotype-based analytical approaches (7, 11, 15), we sought to describe the patterns of haplotype diversity across the span of Europe. Since previous analyses of the genetic structure (24, 25) and haplotypic diversity (26) within Europe, new methodologies utilizing haplotype and IBD-segment data have become available, as have large genomic datasets of European ancestry such as the UK Biobank (UKBB) (27). Therefore, we aimed to address several research questions in European genetics:

1) What sample of European genetics is found in the UKBB dataset?
2) Utilizing chunk-based haplotype methods, how does the genetic structure found within this large European sample match and inform on previous European population genetics research?
3) What is the diversity of demographic histories captured in this sample of European haplotypes?

## Results

### A European Sample from the UKBB.

**Fig. 1.**
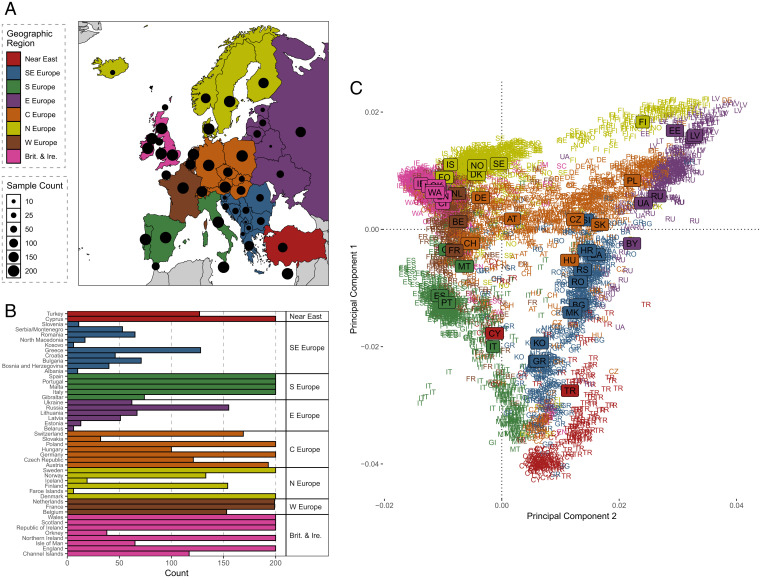
A sample of European structure in the UKBB. (*A*) The number of individuals included from each European country analyzed. Countries are grouped by geographic region; these regions are chosen as a means of group representation and do not necessarily imply historical links. Sample sizes from each region are also shown. Abbreviations are as follows: SE Europe (southeastern Europe), S Europe (southern Europe), E Europe (eastern Europe), C Europe (central Europe), N Europe (northern Europe), W Europe (western Europe), Brit. & Ire. (Britain and Ireland). (*B*) The sample counts for each European region. (*C*) The first two PCs calculated by PLINK of 5,500 European individuals. Individual genotypes are shown by letters that encode the alpha-2 ISO 3166 international standard codes and are color coded according to geographic region. The median PC for each country/region of birth is shown as a label. Plots were generated using the ggplot2 package (65) in the R statistical computing language (59).

Investigating the genetic landscape of Europe, we subsetted individuals form the UKBB (27) who sample European genetic diversity across the continent. This process included selection based on UKBB phenotype data as well as initial genetic analysis and is described fully in *Methods*. In total, 5,550 individuals from 47 European countries/regions (henceforth regions) that spanned the continent were used to investigate the European genetic landscape (Fig. 1).

This European sample refines and expands upon previous work (24). A principal component analysis (PCA) of the allele-frequency-based genetic relationship matrix using PLINK (28, 29) shows that the genetic landscape of Europe is one primarily of gradients. These gradients link genetic regions to one another typically by land but also by sea, as is evidenced in the south of Europe between Italy and Greece and in the Near East populations captured in this dataset. The spread of samples in this genetic space from each region varies (*SI Appendix*, Supplementary Data 2), with samples from most regions forming a primary cluster reflective of their common ancestry. Some regions appear more heterogenous such as Germany or Malta (*SI Appendix*, Supplementary Data 2 and Figs. 2.3 and 2.5), suggestive that a substantial fraction of individuals in the dataset have a recent genetic ancestry that does not match most individuals with the same place of birth label, possibly due to modern economic or other recent migration within the continent. Due to this heterogeneity in birth-location label versus principal component (PC) coordinates, using a separate PCA, we projected our European sample to western Eurasian references from the Human Origins dataset (30). We found agreement between the equivalent labels suggesting the UKBB sample to be representative of the overall structure in Europe.

Interestingly, there appears to be a common cluster of individuals projected onto the same PC space who collectively do not match the ancestry of individuals with their reported place of birth. A substantial number of individuals of Hungarian, Czech, or Russian birthplaces appear to be members of this cluster. Indeed, these individuals separate together on PC six (*SI Appendix*, Fig. 2.9). This suggests a community of individuals with shared common ancestry that is not private to one European country but is more common in samples from Eastern Europe. To further explore the possibility of genetic communities not well captured by country-of-birth-based labels, we sought to cluster individuals based on degrees of haplotype sharing.

### Haplotypes Mirror European Geography.

Conventional haplotype-based approaches (1) struggle to scale to the size of this large sample of European haplotypes. We therefore applied a combination of approaches to organize our European sample into labels based on genetic relationships. We summarized genetic relationships between haplotypes using the “paint” algorithm implemented in the “pbwt” program (31), which scales better at higher sample sizes than the more utilized “ChromoPainter.” Next, we applied the recent network clustering algorithm the Leiden method (32) implemented in the R package “leidenAlg” to cluster individuals iteratively. This allowed a scalable methodology to explore the population structure in this large sample (*Methods*).

**Fig. 2.**
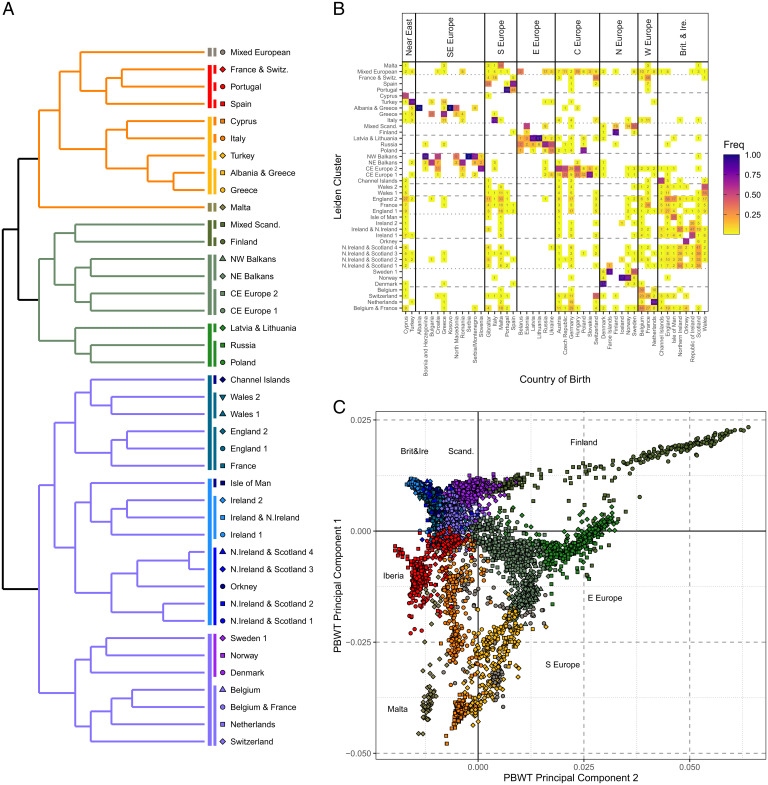
Leiden clustering of 5,500 Europeans from the UKBB. (*A*) The dendrogram of Leiden clusters, grouping them according to their hierarchical relationships. The three main branches are color coded, with additional subdivisions shown as vertical lines. Each of the 41 cluster labels are shown alongside their associated color and shape coding. (*B*) The membership of each of the 41 Leiden clusters. Along the x axis shows country/region of birth, and along the *y* axis cluster membership. The heat map shows the proportion of individuals from each country of birth in each cluster (Freq), and the absolute number. (*C*) The first two PCs of the pbwt paint chunkcounts coancestry matrix. Each point represents the phased genotype of an individual, color and shape coded according to Leiden cluster membership, using the convention shown in *A*. Additional labels are shown to show the broad European region that individuals were born from. Plots were generated using the ggplot2 package (65) in the R statistical computing language (59).

Using the Leiden algorithm, we clustered individuals over a four-step recursive method, allowing us to group related clusters together, which are summarized in the dendrogram output of the Leiden algorithm (Fig. 2). To aid cluster interpretation of the 41 identified clusters, we performed several additional analyses. We recorded region membership per cluster (Fig. 2 and Dataset S1), visualized  the structure captured in the pbwt coancestry matrix by performing PCA (Fig. 2), presented “ADMIXTURE” components stratified by cluster (*SI Appendix*, Fig. 4.1), and compared clusters to Human Origins references (*SI Appendix*, Fig. 4.2). Adapting an approach using the “non-negative-least-squares” function (2) to estimate ancestry profiles, we estimated proportions of haplotype sharing between the 41 clusters to detect recent gene flow and further elucidate relationships between clusters (*SI Appendix*, Fig. 4.3). Finally, we also assessed intercluster distance using total-variance-distance estimates (2) (Dataset S2) and F_ST_ (Dataset S3) using “ADMIXTOOLS2” (*Methods*).

PCA of the pbwt paint coancestry matrix refines our initial PCA based on allele frequency covariances (Fig. 2). It provides more separation of clusters of individuals in the PC space and consistent with similar decompositions of ChromoPainter coancestry matrices (1). We further discuss a comparison of allele-frequency- and haplotype-based PCA results in *SI Appendix*, Supplementary Data 3. Clustering of coancestry sharing results in three broad groups of clusters, as organized into three primary branches of a dendrogram by the recursive R implementation of the Leiden algorithm (Fig. 2). These groups correspond to individuals predominantly born within the northwest of Europe (NW Europe), center and east of Europe (CE Europe), and the south of Europe (S Europe). We highlight interesting information or findings from these regions below; for a full discussion of the clustering and ancestry of these clusters see *SI Appendix*, Supplementary Data 5.

### NW Europe.

The NW Europe branch contains individuals predominantly from Scandinavia, the Low Countries (Netherlands and Belgium), France, Switzerland, the British Isles, and Ireland. Differentiation is low within this branch, as evidenced from an average within-branch fixation index (F_ST_) value of 0.0006 (Dataset S3) and more limited dispersal in PC space (Fig. 2). The NW Europe branch is divided into two main subbranches, separating British and Irish individuals (i.e., those with a British or Irish birthplace) from continental Europeans. We detect 15 clusters of predominantly British or Irish membership, which we attribute to the larger sampling numbers from Britain resulting from treating each country within the United Kingdom as a separate sampling region. We observed a split in Britain and Ireland between the eastern populations of the British Isles (e.g., England and Wales) and the northwestern (e.g., Ireland and Scotland). We report genetic results from the Channel Islands, an archipelago off the northern French coast. Additionally, one cluster of predominant French membership (“France”) groups with English clusters, possibly reflecting gene flow across the channel or a signature of genetic affinity of northwestern France with neighboring Britain (23, 33). Evidence of such admixture is supported in our “nnls” analysis, where France is a mixture of “England 2” and another French membership cluster, namely, “France & Switz.”

Forming an outgroup to the rest of the branch, French, Swiss, Belgian, Dutch, and Scandinavian individuals are branched together, with subbranches separating Scandinavian countries (including Denmark) from the others. All 17 Icelandic individuals sampled are branched with Norwegians, which we attribute to the small sample of Icelandic individuals. In the nnls analysis we observe that the Netherlands and Denmark clusters show evidence of haplotype sharing consistent with their geographic proximity.

### Central-Eastern Europe.

The CE Europe branch contains the following three subbranches: NE Europe (with Baltic, Polish, and Russian membership), CE Europe (with membership from the north of the Balkans and the center and east of Europe), and Finland as an outgroup. Consistent with previous observations (34, 35) Finland shows evidence of isolation from other European regions, projecting away in PC space and showing high differentiation in F_ST_ and total variance distance (TVD) values (Datasets S2 and S3). The majority of Estonian individuals are placed in the “Mixed Scand.” cluster that also groups Swedish, Norwegian, and Finnish individuals who project between Finland and the rest of Scandinavia.

Individuals from the Baltic countries Latvia and Lithuania are clustered together. Estonian individuals project intermediately between Finnish and Baltic–Russian individuals in PC space (Fig. 1), suggesting Finish to Baltic gene flow. Grouped with the “Latvia & Lithuania” clusters are two clusters of predominantly Polish and Russian membership, respectively, forming a cline from east to west (Fig. 2).

The CE Europe branch bridges northeastern and western Europe in PC space (Fig. 2), containing two clusters, namely, “CE Europe 1 and 2.” The latter groups individuals from more western countries and the former more eastern, apparently reflecting the east to west cline in central Europe. This gradual cline in central European genetics (Fig. 2) highlights the need for analyzing continuous genetic data concurrently with clustering analyses for full context of such genetic structure.

The two clusters of northern Balkans membership include 246 of the 448 individuals from the Balkans/SE Europe region (which we additionally associated Romania with). Cluster membership is predominantly from countries north of Albania/Greece/North Macedonia—suggesting a north/south divide on the peninsula that is echoed in PCA (Fig. 2). The “NW Balkans” and “NE Balkans” clusters demonstrate a further geographic cline from east to west, with the former grouping Croatian, Bosnian and Herzegovinian, and Serbian individuals and the latter Romanian and Bulgarian. Our nnls analysis models more southern ancestry (proxied by Greek individuals) in the NE Balkans cluster than the NW (*SI Appendix*, Fig. 4.3).

### Southern Europe.

Leiden clustering yields three broad groups of clusters with southern European membership, as follows: one grouping individuals born around the eastern Mediterranean (i.e., Italy, Greece, Turkey, and Cyrpus), the Iberia Peninsula (Spain and Portugal), and finally two outgroup clusters (“Mixed European” and “Malta”).

Most sampled Greek individuals form a single cluster that in the nnls analysis is modeled as a mixture of haplotypes from neighboring southern clusters such as “Italy” and “Turkey,” but as well haplotypes from the north from NE Balkans. We observe a smaller cluster grouped with the “Greece” cluster on the dendrogram (Fig. 2) that contains all sampled Albanian individuals and also projects separately to Greece in PCA; this finding is suggestive of an additional structure that we explore in our IBD-based analyses below. Elsewhere in this branch are the majority of Cypriots, Turkish, and Italian samples grouped into their own three respective clusters. In Italy, our clustering approach does not resolve the north–south clustering previously observed (4). In the Iberian branch, we group individuals from Spain and Portugal into their own respective clusters that is consistent with previous findings (3), although the “Portugal” cluster contains approximately a quarter of sampled Spanish individuals. In addition, a cluster of mixed French and Swiss membership (France & Switz.) is grouped with Spain and Portugal, appearing to group individuals with haplotype sharing between Switzerland, Italy, France, and Spain in the nnls analysis (*SI Appendix*, Fig. 4.3).

We report to our knowledge the largest sample of dense genome-wide genotypes from the small Mediterranean archipelago of Malta (*n* = 200). In PCA, these are distributed along a gradient along PC seven, forming three clusters. The first contains Maltese individuals who projected separately from the rest with British samples. The other two project away from these Maltese samples, with one equidistant between the British–Maltese individuals and another of sole Maltese membership in PC space (*SI Appendix*, Supplementary Data 6). Our Leiden clustering groups these latter 2 groups of samples into 1 cluster of 79 individuals and the former into clusters of predominant British membership. These results suggest recent western European gene flow into Malta, with the intermediate PCA cluster of Maltese individuals consistent with first-generation offspring of ancestral Maltese and western European parents. Nevertheless, we identify a core cluster of Maltese membership who project onto the same PC space as Human Origin references and with who we investigate the Maltese demographic history below.

Finally, we observe that Leiden clustering identifies a cluster whose membership includes the individuals who separate out along PC six of the PLINK-based PCA. As describe above, these individuals have a mixture of European birthplaces, with a slight bias toward central or eastern Europe. Previous work has shown evidence that the British Jews have been sampled by the UKBB (36). To test whether the membership of this cluster included substantial Jewish ancestry, we utilized references from the Human Origins dataset performing *f*-statistic tests of allele sharing (37) (see *SI Appendix*, Supplementary Data 7) and found strong evidence that this cluster indeed reflects a community with Ashkenazi Jewish ancestry.

### Footprints of European Demography.

**Fig. 3.**
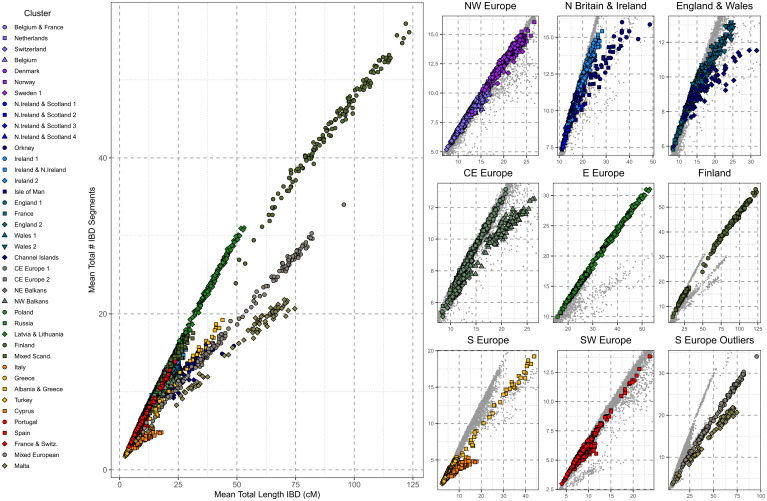
Patterns of within-cluster IBD sharing in the UKBB. (*Left*) The individual mean total length of IBD segments shared with another individual from the same Leiden cluster versus the mean number of IBD segments shared with individuals placed in the same cluster. Individual cluster membership is indicated by symbol/color designation. (*Right*) The values of the panel on the *Left*, showing groups of Leiden clusters separately to highlight subtle regional differences in Europe. Symbol/color designation is the same as the panel on the *Left*. Plots were generated using the ggplot2 package (65) in the R statistical computing language (59).

**Fig. 4.**
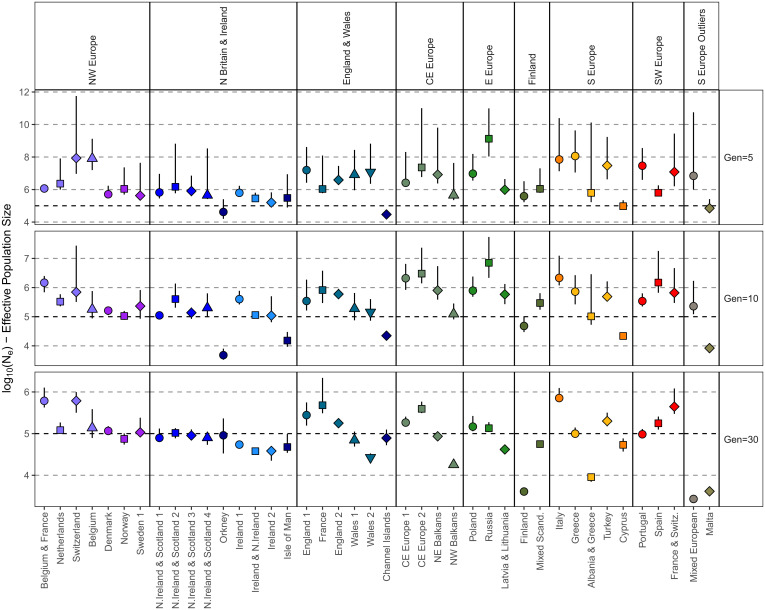
Historical population sizes of different European regions. For each group of related Leiden clusters, the point log_10_ N_e_ estimated by IBDNe is shown for 5, 15, and 30 generations ago. Clusters are indicated by symbol/color designation, and error bars show the lower and upper 95% confidence intervals obtained with bootstrapping. Plots were generated using the ggplot2 package (65) in the R statistical computing language (59).

**Fig. 5.**
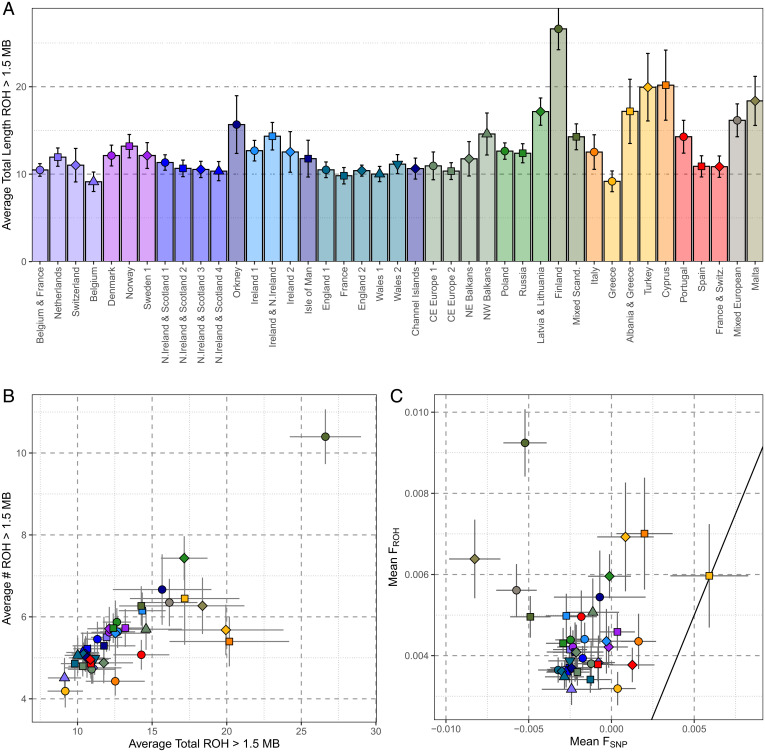
Measures of inbreeding differentiate European genetic histories. (*A*) The per-Leiden cluster; the mean total length of autosomal ROH was >1.5 Mb. (*B*) The average total length of ROH was >1.5 Mb versus the average number of ROH for each Leiden cluster, differentiating the burden of long/short ROH in each cluster. Error bars show 95% confidence intervals. (*C*) The mean *F_ROH_* and *F_SNP_* values for each Leiden clusters with 95% confidence intervals in error bars. Mean *F_ROH_* is an estimate of the total inbreeding relative to an unknown base generation. Mean *F_SNP_* is an estimate of inbreeding in the current generation, with *F_SNP_* = 0 indicating random breeding, *F_SNP_* <0 indicating inbreeding avoidance, and *F_SNP_* >0 indicating inbreeding. Thus, 1) points along the x-axis show excess homozygosity not explained by ROH (caused for example by admixture or excess allele frequency drift compared to coanalyzed samples), 2) points along the *y* axis indicate that homozygosity is caused by historical small population size rather than consanguinity, and 3) points along the solid diagonal line indicate that all excess population homozygosity can be accounted for by ROH.

Having identified genetically homogenous clusters of individuals, we sought to address our third research question pertaining to the landscape of demographic histories within Europe. We leveraged IBD-segment sharing to investigate both the strength and age of isolation (Fig. 3) and the population sizes of each of the clusters over the last ∼2,000 y (*Methods*) (Fig. 4 and *SI Appendix*, Figs. 8.1–5). Complementing these IBD-based approaches, we estimate inbreeding coefficients (*F_ROH_* and *F_SNP_*) to explore the evidence of small-effective population size versus endogamy (Fig. 5).

We first differentiated demographic histories characterized by long IBD-segments shared within populations (for example, recently isolated or practiced endogamy) from those characterized by large numbers of short IBD-segments (for example, a historical bottleneck). For individuals within the same cluster, we plotted the per-individual mean total length of IBD versus the mean number of IBD segments shared (Fig. 3), showing the overall distribution of such values across all clusters (Fig. 3, *Left*) and focusing on subbranches of related clusters (Fig. 3*B*). Generally, we confirm a broad south–north gradient of increasing haplotype sharing in Europe. Individuals of Finnish ancestry present some of the highest levels of within-population IBD sharing in our sample of European haplotypes and is predominated by sharing of short IBD-segments. These Finnish results are consistent with a historical bottleneck and previous genetic observations (38, 39), as well as one of the lowest estimated historical N_e_ in our analysis (Fig. 4) and average coordinates of the inbreeding coefficient that is the proportion of the genome covered by ROH > 1.5 Mb (*F_ROH_*), and the inbreeding coefficient that is measured by the observed versus the number of expected homozyotes (*F_SNP_*) for the Finnish clusters (Fig. 5).

Although S Europe trends toward a larger historical effective population size (N_e_) and low levels of haplotype sharing (e.g., within Italy), there are notable exceptions. Maltese genetic differentiation is expanded upon in IBD-segment analysis (Fig. 3) and agrees with previous IBD estimates from a smaller Maltese sample (40). Malta has a slightly lower average within-cluster total IBD length than Finland, although the average Maltese IBD-segment is longer (Malta IBD segment = 3.17 cM, Finland = 2.07 cM), suggesting a more recent source of this elevated sharing. These results are matched by low historical N_e_. *F_ROH_* and *F_SNP_* analysis show that autozygosity is consistent with a historically small N_e_ rather than consanguinity in Malta.

Within SE Europe, both Turkey and Cyrpus exhibit elevated haplotype sharing (Fig. 3), as well as a lower historical N_e_ (Fig. 4) and evidence of modest consanguinity (Fig. 5). The IBD sharing profile of the Albania and Greece cluster supports evidence of isolation, with elevated haplotype sharing that is equivalent to that found in northeastern Europe (Fig. 2). Albania and Greece also present a consistently lower N_e_ than Greece, and *F_ROH_/F_SNP_* results are consistent with isolation. Elsewhere in SE Europe, we observe NW Balkans presenting slightly longer within-cluster IBD segments than NE Balkans, which is matched with a consistently lower N_e_ and elevated ROH—suggestive of a smaller population than the northeast of the Balkans or neighboring central Europe to the north. Interesting, we find that a subset of Spanish individuals who present elevated within-cluster IBD-segment sharing differ with those of most in Spain (Fig. 3). In a focused analysis (*SI Appendix*, Supplementary Data 9), we conclude that these represent a distinct community or population clustered with other Spanish individuals who nevertheless exhibit elevated haplotype sharing consistent with isolation. These individuals project from other Spanish individuals in PC six (*SI Appendix*, Supplementary Data 9) and when projected on top of Human Origin references project toward “Spanish North” or “Basque” references (*SI Appendix*, Fig. 4.2).

Continuing previous observations of elevated haplotype sharing in island populations, we confirm previous signatures of isolation in island communities in northern Britain and expand with more results. We observe increased haplotype sharing of the British archipelago communities of Orkney, the Channel Islands, and the Isle of Man (Fig. 3) that is consistent with previous observations from Orkney (2), showing results from the Channel Islands and expanding upon previous analyses of the Isle of Man (9). These footprints appear to be more pronounced in Orkney, with a smaller N_e_ 10 generations ago (Fig. 4), as well as slightly longer IBD segments than those shared within the Channel Islands (Orkney IBD segment = 2.18 cM, Channel Island segment = 1.86 cM, Isle of Man segment = 1.90 cM). While Icelandic individuals do not form a private cluster (Fig. 2), there is evidence of elevated IBD sharing consistent with previous observations of homogeneity (41). We observe an increase of the total length of IBD and number of IBD segments between “Norway” Icelanders (i.e., Icelandic individuals placed in the cluster Norway) (45.9 cM and 20 segments, respectively) compared to that observed between Norway Norwegians (22.6 cM and 14 segments). This difference was significant both for total length of IBD (Mann–Whitney *U*, *P* = 4.5 × 10^−11^) or number of segments (Mann–Whitney *U*, *P* = 1.1 × 10^−10^).

Lastly, in an analysis of country-of-birth versus PCA of genetic relationship matrices, and network-based clustering methods, we have identified a community of individuals sampled from the UKBB with evidence of Ashkenazi Jewish ancestry. An analysis of haplotype sharing patterns supports a population of increased haplotype sharing that is intermediate between our Finnish and Maltese profiles (Fig. 3), with a low historical N_e_ 30 generations ago that has expanded within the past 10 generations (Fig. 4) and an increase in homozygosity (Fig. 5). In a focused analysis, we show that this cluster contains two broad groups of individuals, as follows: one with elevated haplotype sharing, with more and longer IBD and ROH detected, and another with a mixture of ancestries reflective of individuals with a recent admixture outside of the community and a generally higher historical N_e_. This cline of elevated haplotype sharing is captured by PC three of the PCA of the pbwt paint coancestry matrix.

## Discussion

Utilizing a subset of 5,500 individuals from UKBB (27), we have demonstrated that the dataset contains a wealth of ancestries not limited to that ancestral to Britain or Ireland, which may be of interest to researchers interested in communities with non-European ancestry potentially sampled in the UKBB. We have leveraged a large European sample to present an updated map of the genetic structure present across the continent and provide insights into the footprints of demographic histories within Europe. While our haplotype-based clustering analysis divides this sample into three main branches of S, CE, and NW Europe, lower dimensional analyses reveal that the genetic landscape of Europe is one of clines. Undoubtably, focused studies on individual European populations have revealed illuminating insights into fine-scale structure and population histories of those regions (2, 4, 7–9, 23, 33, 38). This analysis of the overall structure of Europe using a Europe-wide sample has been able to show the connected haplotypic landscape of Europe together, as well as leveraging IBD sharing and ROH to demonstrate European genetic histories in parallel.

This work has also demonstrated the potential utility in biobanks like the UKBB in exploring the genetics of populations outside of their sampled region (i.e., the British Isles). Worldwide ancestries have been shown to be captured by the UKBB (42), and this work has demonstrated that the selection of key phenotypes with an initial analysis of global ancestries can provide an informative sample of a continental group. The comparison with ancestry-ascertained references from the Human Origins dataset (30) demonstrates good overlap between the distribution of ancestries captured, although it is possible that we are not able to account for subtle biases in fine-scale regional structure. Furthermore, we expect some bias in the ancestry of individuals from each country who were able to move to the United Kingdom and participate in the UKBB, which may further bias these subtler local regional structures.

Our results have implications for genetic mapping, for example via the characterization of genetic isolates. The haplotype profile displayed by Malta is significant in this context. The footprints of isolation in the primary Maltese cluster are comparable to more documented isolates such as Iceland, Finland, or Orkney. We are confident that our results are representative of the overall Maltese genetic profile for several reasons. In sampling individuals from Malta, we observe three groups of individuals in PCA, as follows: one of individuals placed mainly in clusters of British membership, one forming the majority of the *Malta* cluster, and another projecting in between the former two. We therefore already identify recent migrants to the island who do not represent the historical ancestry there. The collective genetic profile of the Malta cluster is also consistent with previous literature, where Maltese uniparental data (43) and limited autosomal STR data (44), as well as analysis (40) of a small sample of Maltese SNP-array genotypes from the Human Origins dataset (30) have shown evidence of an island genetic isolate. Indeed, these Human Origin Maltese genotypes colocate with our sample in the projected PCA (*SI Appendix*, Fig. 4.2). Our results, in the context of disease mapping work highlights the potential of this isolate community in genetic mapping efforts. As similarly argued recently by Borg et al. (45), identifying such isolates for study has the potential to expand captured rare, community-specific disease variation and aid genetic disease research.

Our result highlights other communities relevant to genetic mapping efforts, some that are well established in the literature such Orkney and Shetland (20, 21, 46, 47) or the Basque (48–51) and some novel, e.g., the Channel Islands. The Channel Islands are an archipelago of isles off the northern coast of France and are a British dependency. Arguably, our sampling scheme, i.e., birthplace, is not specific to the islands compared to other previous British sampling ascertainments (2, 9), and this is demonstrated where individuals with a Channel Island birthplace are found in a number of British clusters (Fig. 2). Nevertheless, we do identify a cluster of predominant Channel Island membership that is distinct in IBD analysis. It presents a profile like that in Orkney, suggesting there is a community of reduced haplotype diversity on the islands, which warrants further study.

We improve sample coverage in SE Europe where sampling has been lower than other European regions in previous analyses (52–54). Our results demonstrate a north–south cline in this region at the crossroads of S Europe, the Near-East, and NE Europe. This cline is associated with higher haplotype sharing in the north and haplotype affinity to Italy in the south. Our observations expand upon and agree with previous work that included Balkans genotypes (54), observations of a genetic cline on the peninsula (52), and relationship with northern European genetics (53). Greece in the south appears genetically distinct in our analysis, although this may be driven by the higher sample size of Greek samples compared to other regions with the peninsula. Interestingly, we capture a community of relatively isolated individuals (“Albania & Greece”), with haplotype sharing equivalent to Orcadian or Mixed European individuals. This small cluster (*n* = 38) consists of Albanian, Kosovan, and some North Macedonians. Whether this is a specific immigrant community sampled by happenstance in the UKBB or a feature of the region is unclear, and expanded sampling within this region may further elucidate how varied the haplotype landscape of this region is.

Elsewhere in S European genetics, we have studied a genetic community of individuals that we denote as Mixed European. Although we discuss in more detail in *SI Appendix*, Supplementary Data S7, to summarize, we show using *f*-statistics (37) that these individuals share ancestry with Ashkenazi Jewish references from the Human Origins dataset as well as projecting onto the same PC space (*SI Appendix*, Fig. 4.2). The demographic profile from haplotype sharing and estimated historical N_e_ of individuals within this cluster matches well with a proposed demographic model from a previous study of European Ashkenazi Jews (55) with a bottleneck 25 to 35 generations ago with a subsequent period of population expansion. This model included admixture between southern European and Middle Eastern sources and latter Eastern European sources (55), which match our nnls sharing patterns of IBD (*SI Appendix*, Fig. 4.3).

Overall, our work has demonstrated a utility of large cosmopolitan biobank studies in providing an informative continental sample of European genotypes. We have leveraged this to expand the map of the European genetic landscape and show genetic signatures of interest to geneticists. The use of joint analysis of both chunk- and IBD-based haplotype analysis shows that they complement each other to interrogate the population structure and demographic profile of different communities on the continent. This work raises the possibility of similar analyses of non-European communities within the UKBB, as well as highlighting the need for focused genomic analysis on European regions not typically captured by large datasets to provide fine-scale insights into their genetic history and current stratification of genetic variation.

## Methods

### Identification of European Sample of UKBB.

To select a European sample of genotypes from the UKBB, multiple filtering steps were carried out by firstly selecting individuals based on phenotype codes provided in the UKBB. Phenotype codes are indicated with a F.X coding, where X is a numerical code. Further data coding within each of these phenotypes are indicated with a C.X coding, where X is a numerical code. We first selected individuals with an ethnicity (F.21000) that was either “White” (C.1), “White British” (C.1001), “White Irish” (C.1002), “any other white background” (C.1003), or “other ethnic group” (C.6). The proportions of these ethnicity labels in each European country of birth group label and Leiden cluster are shown in *SI Appendix*, Supplementary Data 1.

These individuals were further filtered based on a birthplace (F.20115) from Europe (C.300-C.400), excluding countries from central Asia or Caucus (Armenia (C. 303, *n* = 5), Azerbaijan (C. 305, *n* = 6), Georgia (C.319, *n* = 4), and Kazakhstan (C.327, *n* = 15)) and also excluding United Kingdom (C.354) and Irish (C.356) birthplaces. In addition to these individuals, we separately identified individuals with a British or Irish birthplace (F.1647), selecting from England (C.1), Wales (C.2), Scotland (C.3), Northern Ireland (C.4), and the Republic of Ireland (C.5). Furthermore, we identified additional individuals with a geographic birth coordinate (F.129, and F.130, Easting and Northing) that corresponded to the island(s) of Orkney (11e5 < Northing < 12.5e5, and 4e5 < Easting < 5e5), Isle of Man (2e5 < Easting < 2.5e5, and 4.5e5 < Northing < 5e5). Lastly, we randomly down-sampled countries to 200 individuals if their sample size was larger to retain comparable sample sizes between regions.

With this dataset, we performed initial PCA using PLINK (28, 29) to identify ancestry outliers. With PLINK and raw single nucleotide polymorphism (SNP) genotypes (F.22418), we filtered for autosomal genotypes, removing stand-ambiguous SNPs (A/T, G/C); removing SNPs with missingness of >5%, minor allele frequency (MAF) of <2%, and Hardy–Weinberg equilibrium (HWE) of *P* <1e6; removing individuals with missingness of >5%; and removing one individual from a pair of individuals related closer than third degree with KING (56). We calculated PCs on a set of SNPs pruned with respect to linkage disequilibrium using the PLINK command –indep-pairwise 1000 50 0.2. With these PCs, we manually identified and removed overall outlier samples in European samples indicative of non-European ancestry.

This left 5,500 individuals with a common set of markers that were non-strand-ambiguous SNPs with missingness of <5%, MAF of >2%, and an HWE *P* >1e6 (482,591 markers).

### European Population Structure.

To explore the sampled population structure of Europe from 5,500 individuals from the UKBB, we initially performed PCA using PLINK (28, 29) and estimated ancestry components using the model-based maximum likelihood method of ADMIXTURE (57). In each analysis, we used the 5,500 individuals and 204,652 SNPs that had been pruned with regard to linkage disequilibrium, i.e., they were independent markers. In ADMIXTURE analysis, we modeled every individual as a mixture of *k* ancestral components (from 2 to 7) over 10 replicate runs, choosing the replicate with the highest log-likelihood and the lowest cross-validation error.

Comparing the ancestries of our European sample from the UKBB, we coanalyzed these with population references from the Human Origins dataset (30). We selected 905 “West Eurasians” from the Human Origins dataset, selecting ancestries within and adjacent to Europe for further context. We merged the genotypes of the 4,920 UKBB Europeans and the 905 Human Origins West Eurasians, using the SNPs common to both datasets, and using PLINK (28, 29), we selected individuals and SNPs with <5% missingness, a MAF of >2%, and a HWE *P* >1e-6. We additionally pruned for linkage disequilibrium using the plink command –indep-pairwise 1000 50 0.2, leaving a final common SNP count of 46,173 variants. We performed PCA, projecting UKBB Europeans onto the genetic variation of the Human Origins West Eurasians in PLINK using the –within and –pca-cluster-names in conjunction with the –pca command.

### Chunk Haplotype Analysis.

Using the dataset of 5,500 Europeans and 482,591 markers, we phased haplotypes with Beagle v5.1 (58) using default parameters and a recombination map of human genome build 37. These phased haplotypes were subsequently used for both pbwt (31)-based and IBD-based analyses. Due to the large sample size, “fineSTRUCTURE” clustering of the CHROMOPAINTER coancestry matrix has a significant cost, indeed as does the estimation of the CHROMOPAINTER coancestry matrix, although that is parallelizable. We therefore instead utilized the pbwt (31) program to estimate the coancestry matrix in a more scalable fashion and then utilized network community clustering approaches to group our European sample into genetically related communities.

Using the phased genotypes from Beagle, we calculated the coancestry matrix for each autosome with the pbwt (31) utility’s paint algorithm, setting the number per region as 100 to match default ChromoPainter (1) parameters. To estimate the whole-autosome “chunkcounts” and “chunklengths” coancestry matrices, we summed each of the per-autosomal coancestry matrices together.

With the pbwt paint chunkcount coancestry matrix as the input, we constructed a network graph using the statistical computing language R (59) and the “igraph” (60) package. We constructed a network with every individual’s diploid haplotype as a node and the chunkcount value between each individual as edges. We filtered edges >2 and <25 to down-weight deep genetic relationships as well as recent genetic relationship within the past few generations as an equivalent to previous IBD-based network clustering approaches (12, 13). With this network, we performed Leiden community detection (32) (henceforth “clustering”). The Leiden method has been shown to be faster than the more commonly used Louvain method (12, 13), as well as yielding communities that are more connected, and when used iteratively, the nodes in the identified communities are locally optimally assigned to clusters. We used the “leidenAlg,” an implementation in R, rleiden.community function, using default parameters with the exception of max.depth (we set this to 4 to perform a 4-step recursive clustering process) and min.community.size (we set this to 100 to ensure small clusters of <100 individuals were not attempted to be subdivided).

Visualizing haplotype sharing, we applied PCA to the pbwt paint chunk count coancestry matrix with scripts provided by the authors of *fs* (1). We additionally applied the R (59) implementation of the t-distributed stochastic neighbor embedding (t-SNE) algorithm (61) to the top 10 coancestry PCs using default parameters aside from max_iter = 2,000 (*SI Appendix*, Fig. 4.4).

Quantifying haplotype sharing profiles between Leiden clusters, we utilized a modification of a regression nnls-based method to estimate ancestry profiles (2). Using the pbwt paint chunklengths coancestry matrix as the input, we estimated haplotype sharing profiles for each Leiden cluster. Specifically, we modeled each cluster as a “target” cluster, estimating the proportion of haplotype sharing donated from *P* “source” clusters, where we treated every other Leiden cluster as a potential source. For each target cluster of *N* individuals, we recorded a *X* matrix that has *N* rows and *P* columns, recording the summed chunklengths that each source cluster donates to each target cluster individual. We record a *Y* matrix that records the mean summed chunklength estimates between each source cluster. Using the same nnls adaption as used by Leslie et al. (2), we estimate our equivalent of *β_g_*, the average proportion of haplotypes in an individual in each target Leiden cluster that is most closely related to each source cluster. We show these *β_g_* values in a heat plot (*SI Appendix*, Fig. 4.3). This approach appears to identify the closest neighbors of haplotype identity, as well as recent admixture (*SI Appendix*, Fig. 4.3), and thus does not capture the deep relatedness between all Europeans (62).

To quantify genetic distance between these clusters, we calculated F_ST_ using ADMIXTOOLS2 (63), an R package implementation of *f*-statistics that can also calculate a Hudson estimate of F_ST_. We calculated this between clusters using 204,652 SNPs that were pruned with regard to linkage disequilibrium. We additionally calculated the TVD between clusters using the methodology of Leslie et al. (2) and applying it to the pbwt chunklengths coancestry matrix. This measures the difference between copying vectors of a pair of fineSTRUCTURE clusters.

### IBD Analysis.

We detected IBD segments in our European sample with refinedIBD (v17Jan20.102) (64), detecting IBD segments with a minimum length of 1 cM. Genotyping and haplotype phase-switch errors can erroneously split true IBD segments apart into shorter but adjacent detected segments. Therefore, we used the merge-ibd-segments.17Jan20.102.jar utility to remove such breaks or short gaps between IBD segments, removing gaps that contain at most one discordant heterozygote and less than 0.6 cM. To estimate N_e_, we used the IBDNe program (15), selecting IBD segments >4cM that were shared between individuals belonging to the same Leiden cluster. We ran the IBDNe program, using 100 bootstrap samples to compute confidence intervals. We show the N_e_ estimates for 30, 15, and 10 generations ago in Fig. 4, and show the full curves for each cluster in *SI Appendix*, Supplementary Data 8.

### Measures of Inbreeding *F.*

Exploring the degree of isolation in our European sample, we calculated two estimates of the inbreeding coefficient (*F*), namely, *F_ROH_* and *F_SNP_* (22).

*F_ROH_* is defined as the fraction of the total genome (2,879,248,291 bp) in ROH of >1.5Mb. We detected ROH using PLINK with the following parameters: –homozyg-window-snp 50; –homozyg-snp 50; –homozyg-kb 1500; –homozyg-gap 1000; –homozyg-density 50; –homozyg-window-missing 5; –homozyg-window-het 1. No linkage disequilibrium pruning was performed.

*F_SNP_* is a SNP-based measure of inbreeding in the most recent generation and is an estimate of *F_IS_* (22). We calculated *F_SNP_* using PLINK’s –het command that is an implementation of:

$$F_{\mathrm{SNP}}=\frac{O\left(\text{HOM}\right)-E\left(\text{HOM}\right)}{N-E\left(\text{HOM}\right)}$$

where *O*(HOM) is the observed number of homozygous SNPS, *E*(HOM) is the expected number of homozygous SNPs given the HWE, and *N* is the total number of SNPs. We calculated *F_SNP_* separately for groups of clusters, as follows: NW Europe (Belgium & France, Netherlands, Switzerland, Belgium, Denmark, Norway, Sweden 1), N Britain & Ireland (N.Ireland & Scotland 1, N.Ireland & Scotland 2, N.Ireland & Scotland 3, N.Ireland & Scotland 4, Orkney, Ireland 1, Ireland & N.Ireland, Ireland 2, Isle of Man), England & Wales (England 1, France, England 2, Wales 1, Wales 2, Channel Islands), CE Europe (CE Europe 1, CE Europe 2, NE Balkans, NW Balkans), E Europe (Poland, Russia, Latvia & Lithuania), Finland 1 (Finland), Finland 2 (Mixed Scand.), S Europe (Italy, Greece, Albania & Greece, Turkey, Cyprus), SW Europe (Portugal, Spain, France & Switz.), Mixed European (Mixed European), and Malta (Malta).

## Supplementary Material

Supplementary File

Supplementary File

Supplementary File

Supplementary File

## Data Availability

UKBB genotype data can be accessed from the UKBB through the process specified at https://www.ukbiobank.ac.uk/scientists-3/genetic-data/. The Human Origins genotype data with population labels can be publicly accessed from the David Reich Lab at https://reich.hms.harvard.edu/datasets.
