# Supplementary material for: Revealing the recent demographic history of Europe via haplotype sharing in the UK Biobank
Source: Proc Natl Acad Sci U S A. 2022 Jun 13;119(25):e2119281119. doi: 10.1073/pnas.2119281119 (PMC9233301; doi:10.1073/pnas.2119281119)
Supplement: Supplementary File [file pnas.2119281119.sapp.pdf]

Revealing the recent demographic history of Europe via haplotype sharing in the UK Biobank.

Supplementary Information Appendix

Authors

Edmund Gilbert[1,2]*, Ashwini Shanmugam[1,2,3], Gianpiero L. Cavalleri[1,2,3]*.

Affiliations

1. School of Pharmacy and Biomolecular Sciences, Royal College of Surgeons in Ireland, Dublin.
2. FutureNeuro SFI Research Centre, Royal College of Surgeons in Ireland, Dublin.
3. The SFI Centre for Research Training in Genomics Data Science, Ireland.

* Corresponding Author

Table of Contents

Authors....1
Affiliations....1
Supplementary Data 1 - UK Biobank Self-Reported Ethnicity....3
Supplementary Data 2 – Individual Country Population Structure....4
  Supplementary Data 2.1 - Per Country PCA Distributions....4
  Supplementary Data 2.2 - Principal Component Analysis of UK Biobank Europeans....12
  Supplementary Data 2.3 - Per ADMIXTURE Component Proportions....13
  Supplementary Data 2.3 - Comparison to Human Origins References....14
Supplementary Data 3 – Comparison on Linked and Unlinked Methods....15
Supplementary Data 4 - Genetic Structure of European Leiden Clusters....18
Supplementary Data 5 – Detailed European Genetic Landscape....22
  North-Western Europe....22
  Central-Eastern Europe....23
  Southern Europe....24
Supplementary Data 6 - Malta....25
Supplementary Data 7 - Mixed Europeans....26
Supplementary Data 8 - IBD Ne Curves....31
Supplementary Data 9 - Spain....36

References 39

Supplementary Data 1 - UK Biobank Self-Reported Ethnicity

Whilst sampling European ancestry within the UK Biobank[1], we selected based on European birthplace and self-reported ethnicity, selecting the background which fell under the parent description “White” (F.21000 = 1/1001/1002/1003) or “other” (F.21000 = 6) (see Methods). With comparison to West Eurasian references from the Human Origins[2] by projection principal component analysis (PCA) this reveals a sample of European ancestry across the continent (**SI Appendix Figure S2.11**). We further recorded the proportions of the selected self-reported ethnicity categorises within each sampled country/region of birth (**Supplementary Figure 1.1**), as well as each Leiden[3] cluster subsequently detected (**Supplementary Figure 1.2**).

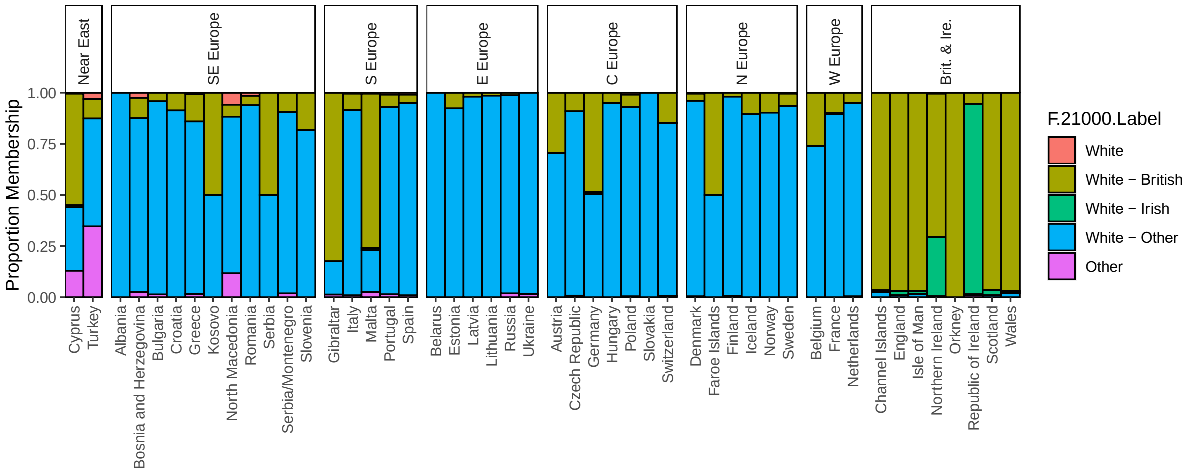

Supplementary Figure 1.1 – Per-country/region of birth proportions for five self-reported ethnicity categories from the UK Biobank. The ethnicity category corresponds to UK Biobank phenotype code F.21000.

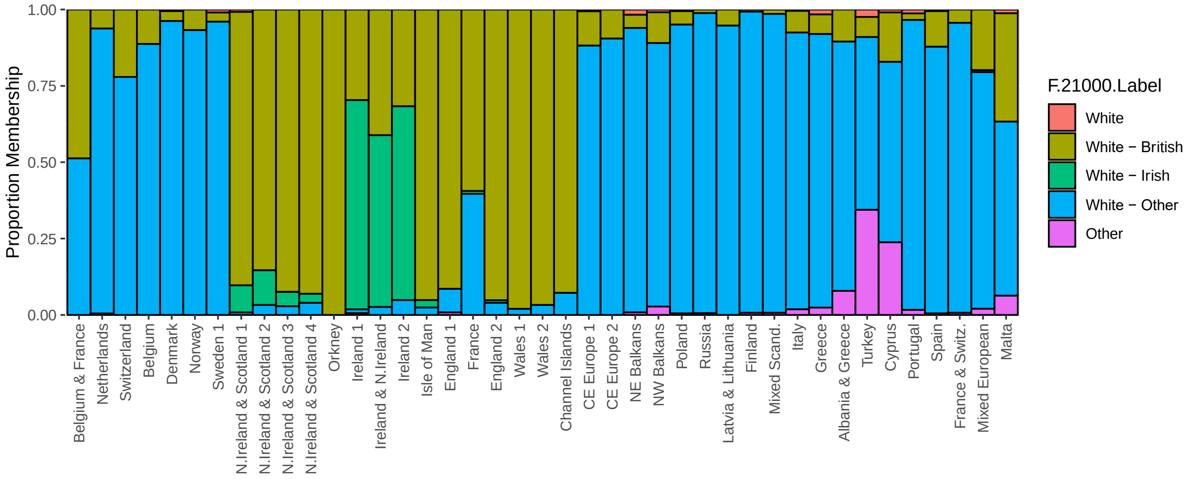

Supplementary Figure 1.2 – Per-Leiden-cluster proportions for five self-reported ethnicity categories from the UK Biobank. The ethnicity category corresponds to UK Biobank phenotype code F.21000.

Supplementary Data 2 – Individual Country Population Structure

Supplementary Data 2.1 - Per Country PCA Distributions

In sampling of UK Biobank[1] participants with European ancestry and birthplace we performed an initial principal component analysis (PCA), investigating the genetic structure in the sample of 5,500 individuals, and the genetic structure sampled in each individual country/region of birth. Below we record the distribution of individuals for each such region, grouped by geographic proximity. Each plot shows the coordinates from the PCA shown in **Figure 1**, highlighting individuals from each individual region in red and every other individual as a small grey circular point.

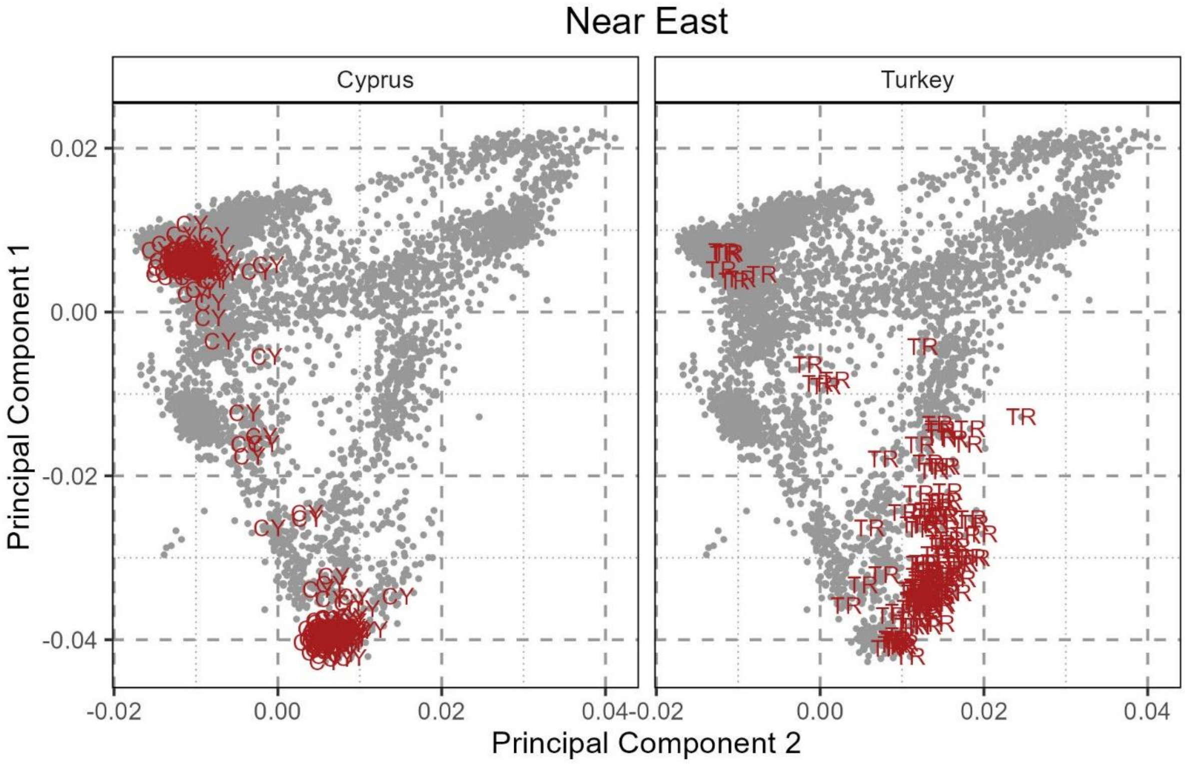

Supplementary Figure 2.1 – Principal Component coordinates of UK Biobank individuals with Cypriot, or Turkish birthplace.

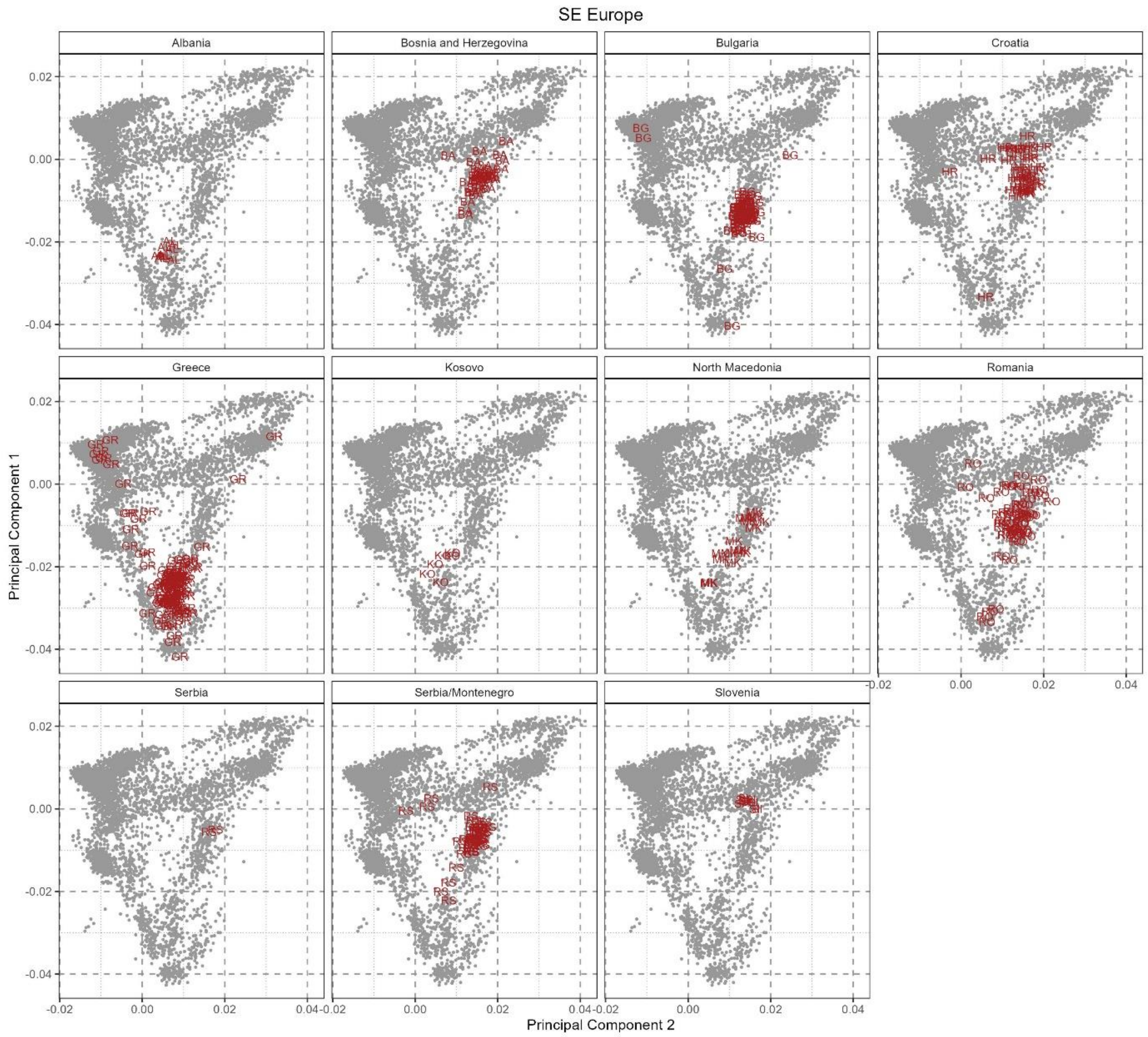

Supplementary Figure 2.2 – Principal Component coordinates of UK Biobank individuals with Greek, Kosovan, North Macedonian, Romanian, Serbian/Montenegro, or Slovenian birthplace.

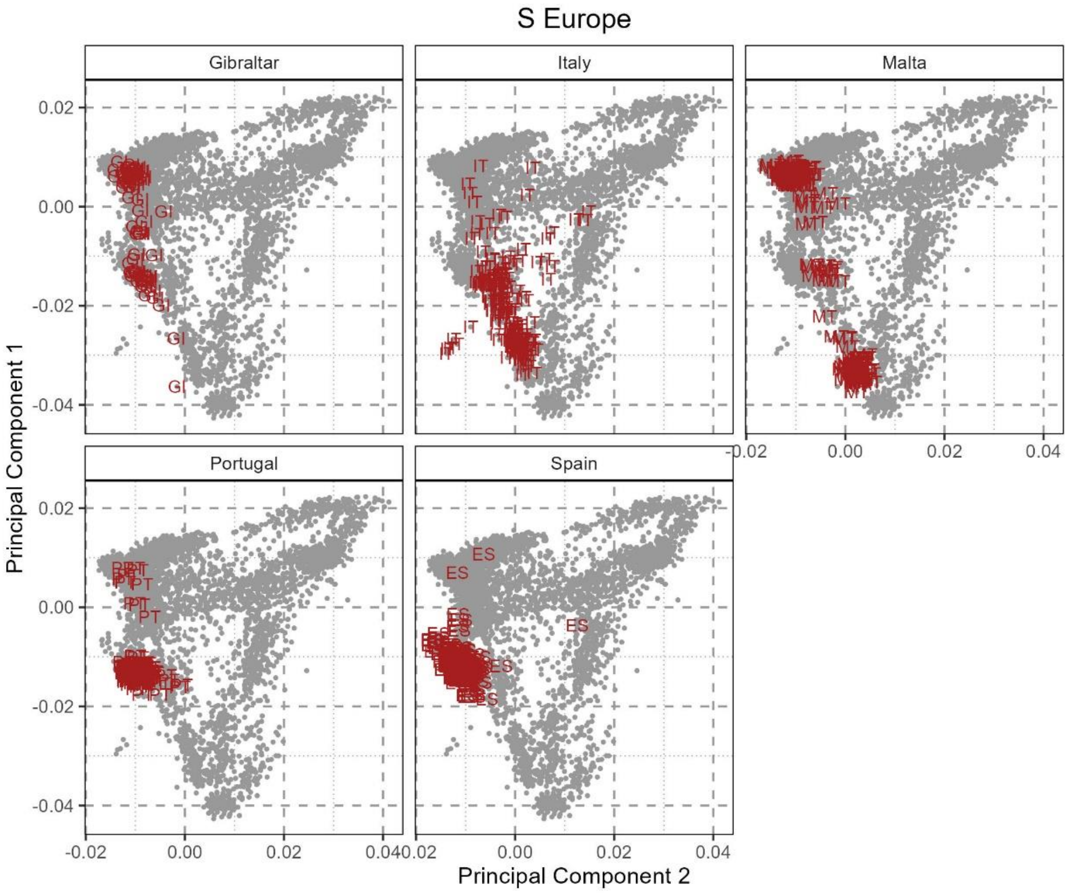

Supplementary Figure 2.3 – Principal Component coordinates of UK Biobank individuals with Gibraltarian, Italian, Maltese, Portuguese, or Spanish birthplace.

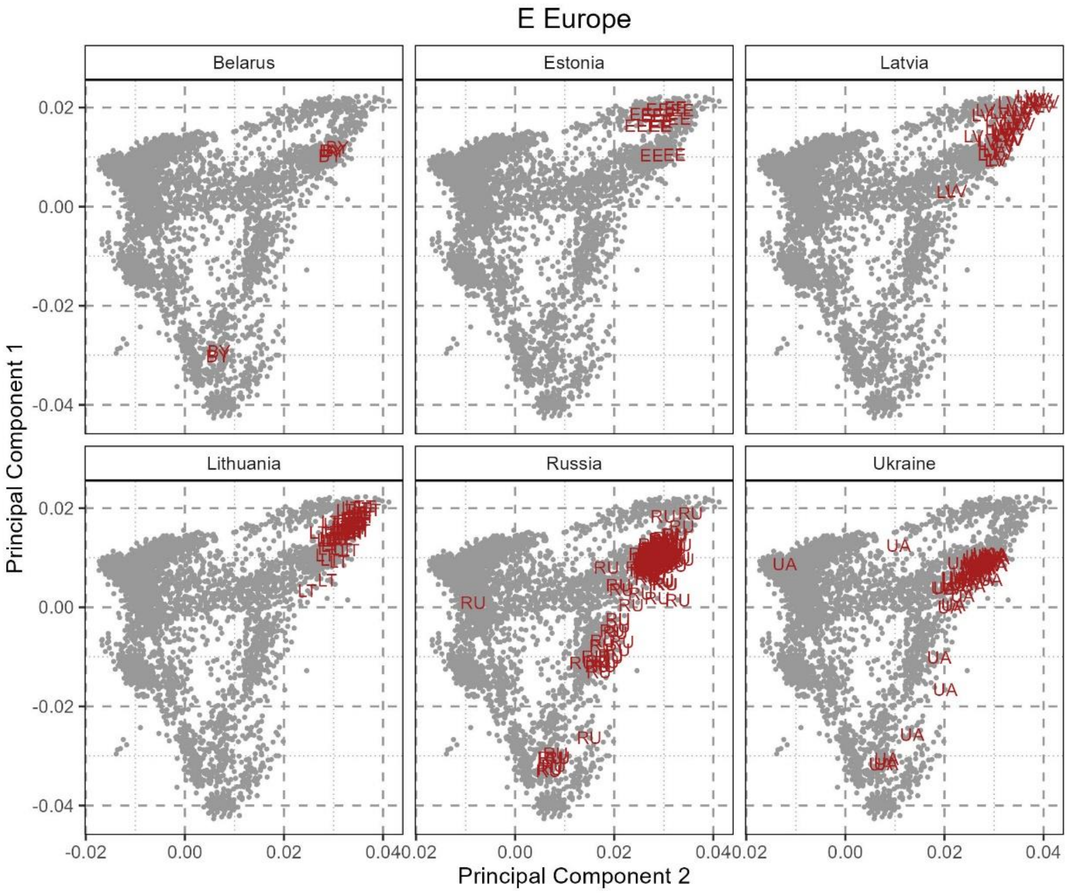

Supplementary Figure 2.4 – Principal Component coordinates of UK Biobank individuals with Belarusian, Estonian, Latvian, Lithuanian, Russian, or Ukrainian birthplace.

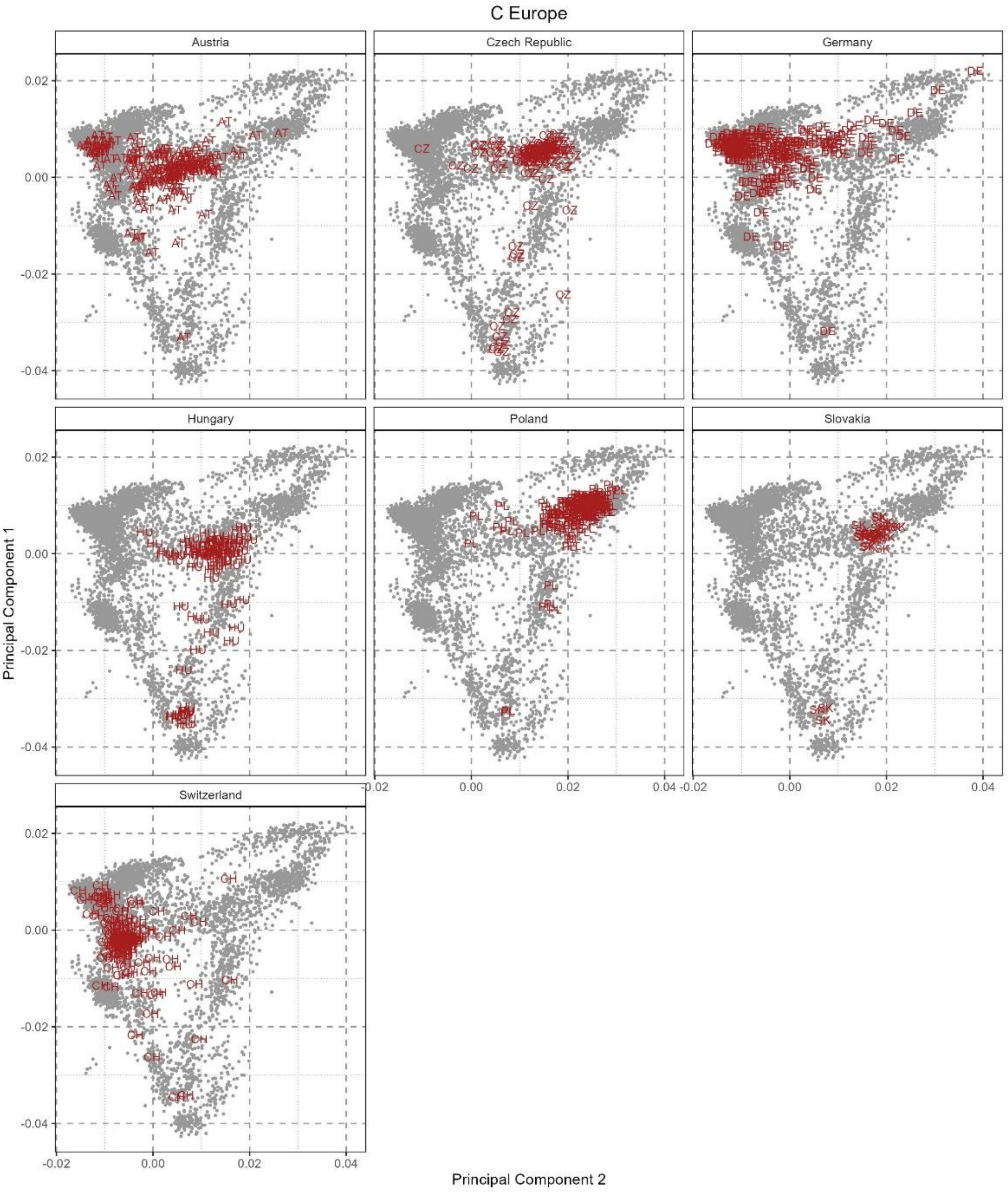

Supplementary Figure 2.5 – Principal Component coordinates of UK Biobank individuals with Austrian, Czech, German, Hungarian, Polish, Slovakian, or Swiss birthplace.

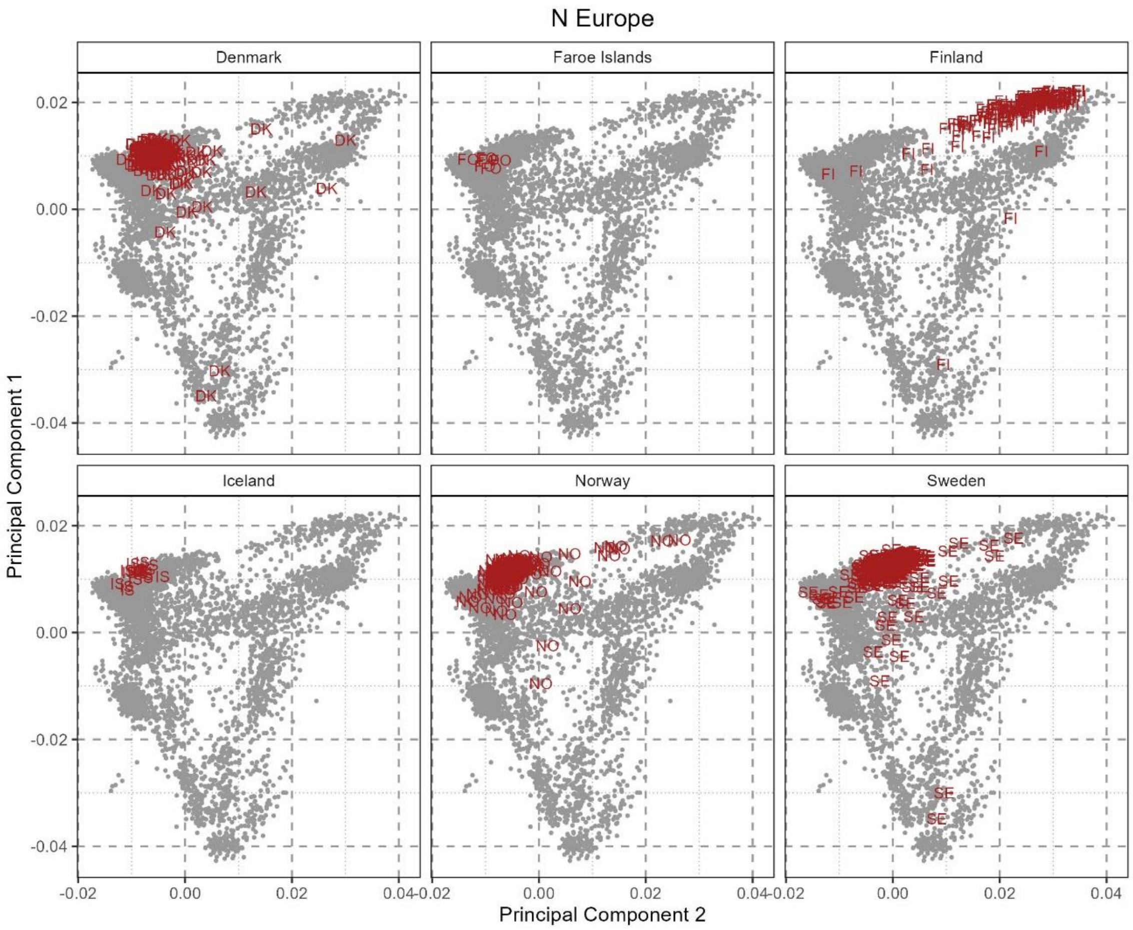

Supplementary Figure 2.6 – Principal Component coordinates of UK Biobank individuals with Danish, Faroese, Finnish, Icelandic, Norwegian, or Swedish birthplace.

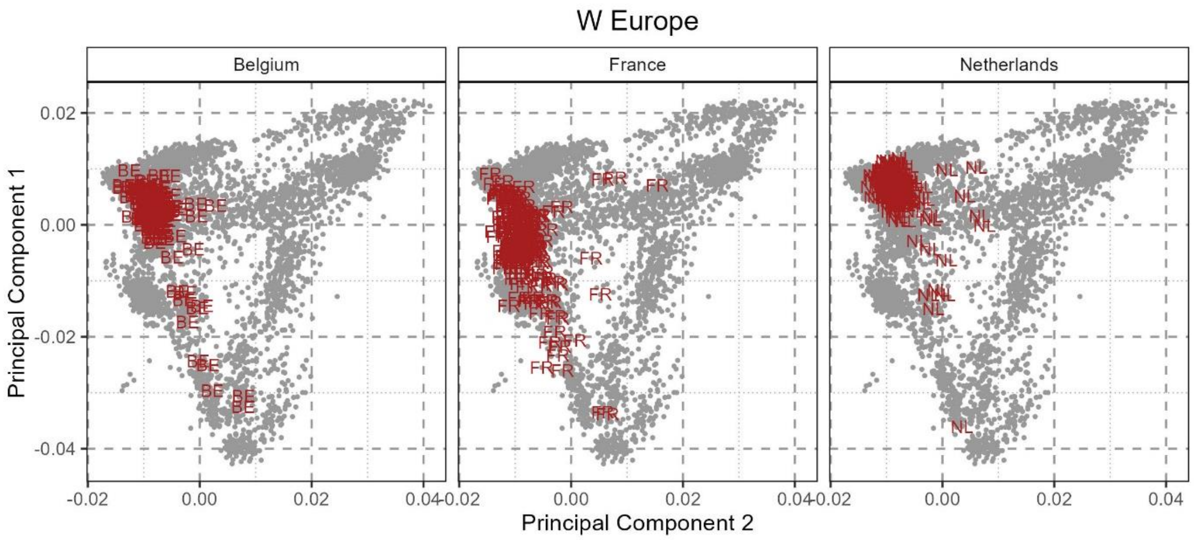

Supplementary Figure 2.7 – Principal Component coordinates of UK Biobank individuals with Belgian, French, or Dutch birthplace.

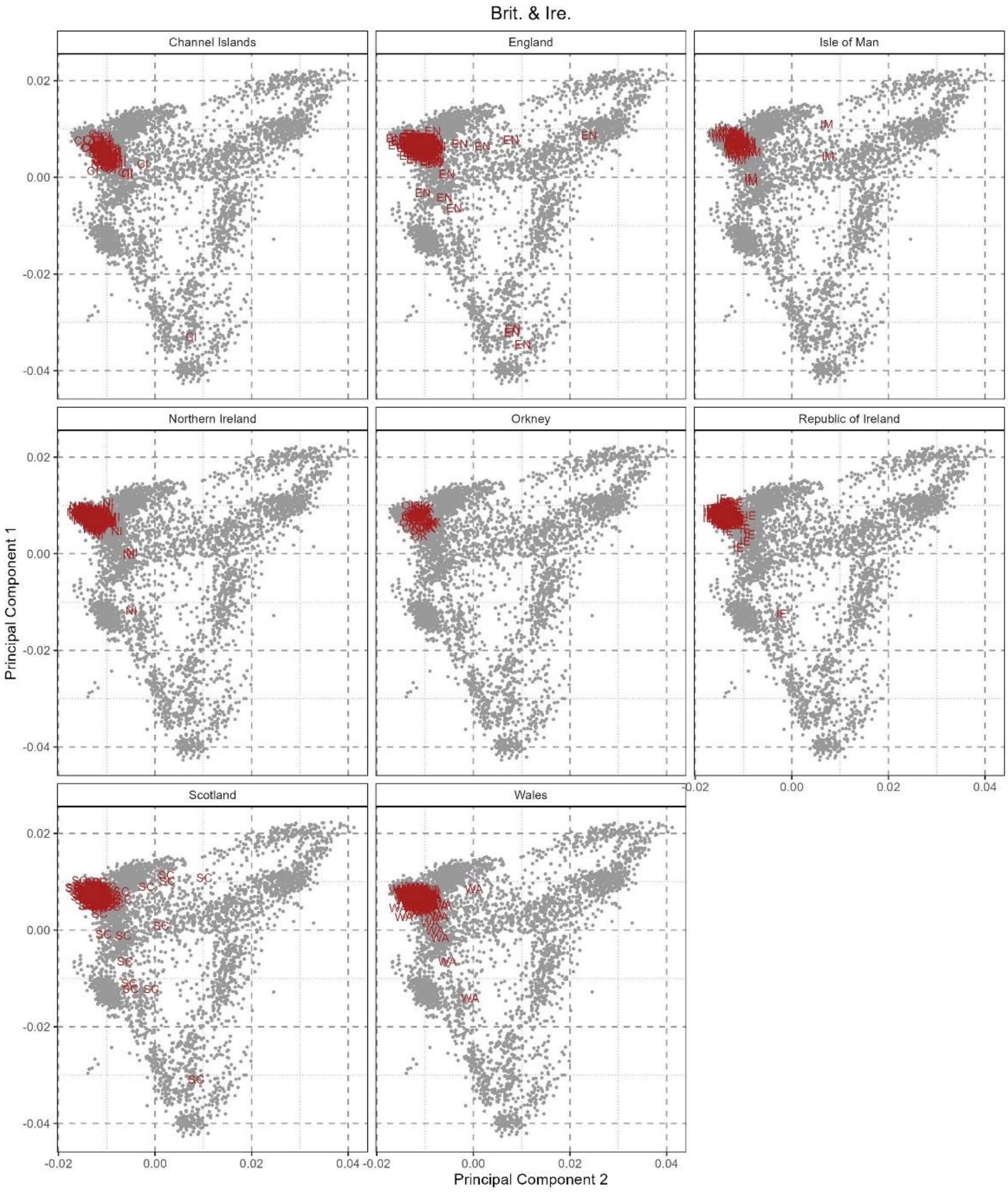

Supplementary Figure 2.8 – Principal Component coordinates of UK Biobank individuals with British or Irish birthplace.

Supplementary Data 2.2 - Principal Component Analysis of UK Biobank Europeans

To supplement Figure 1, we show below the additional principal components three through to ten. These were calculated using the same methodology as those principal components shown in Figure 1, with the same legend and codes for country/region of birth. For each of the four panels, the x-axis principal component is given first and then the y-axis principal component (PC), i.e., for "PC3-PC4", PC three forms the x-axis and PC four forms the y.

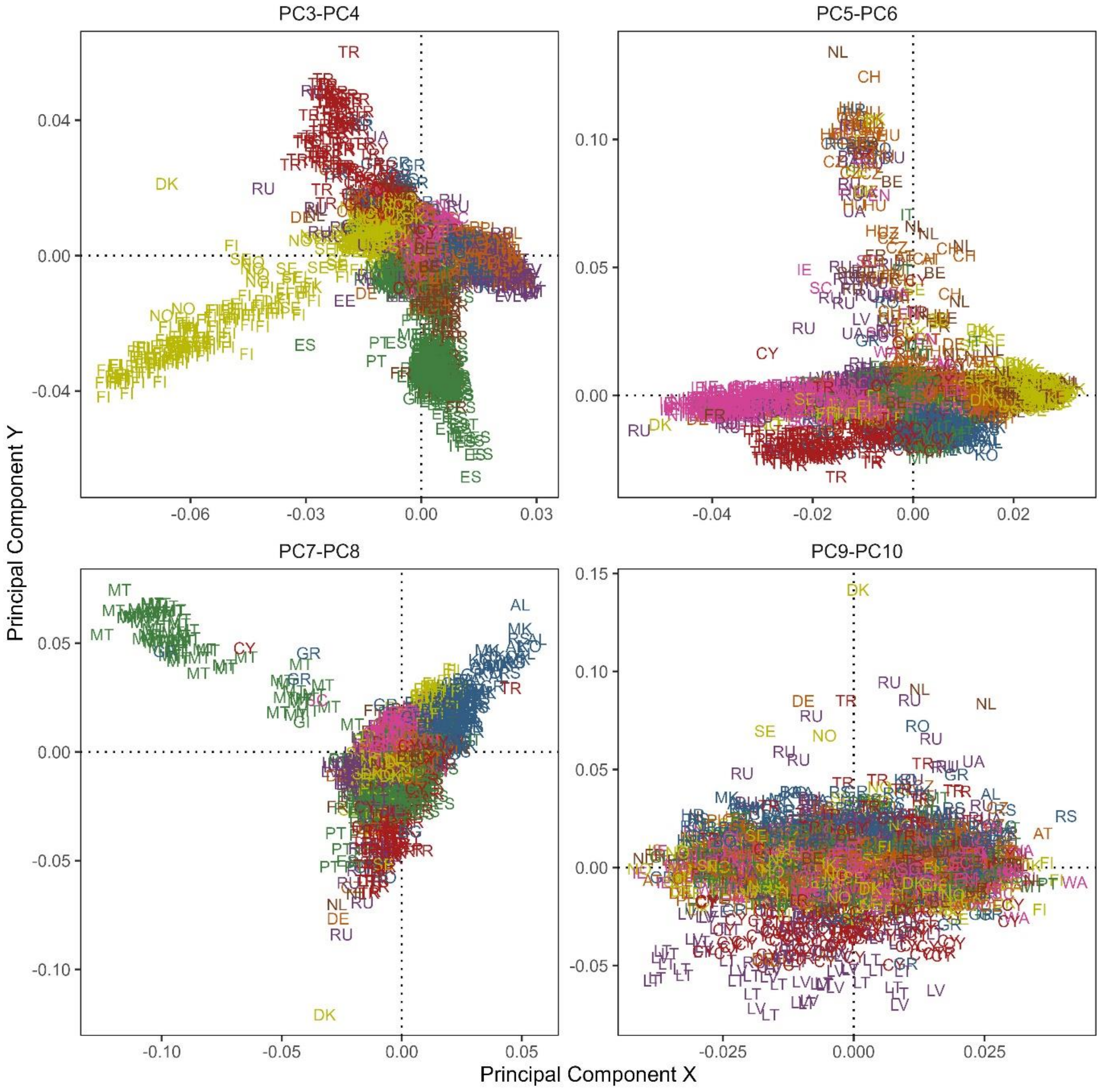

Supplementary Figure 2.9 - Principal component (PC) analysis of 5,500 Europeans from the UK Biobank[1] using PLINK[4,5] across PCs three through ten.

Supplementary Data 2.3 - Per ADMIXTURE Component Proportions

Further, we performed ADMIXTURE[6] analysis on the same individuals and common markers there were used for the PCA using PLINK[4,5] above (see Methods). ADMIXTURE v1.3 is a fast modelled-based maximum likelihood estimation of an individuals' ancestry, assuming *k* ancestral "populations" or components. To explore the genetic structure in the UKBB 5,500 sample we ran ADMIXTURE over *k* values from two to seven, run ten replications for each *k* value and choosing the replicate with the high log-likelihood and lowest cross-error validation score. We show the results below:

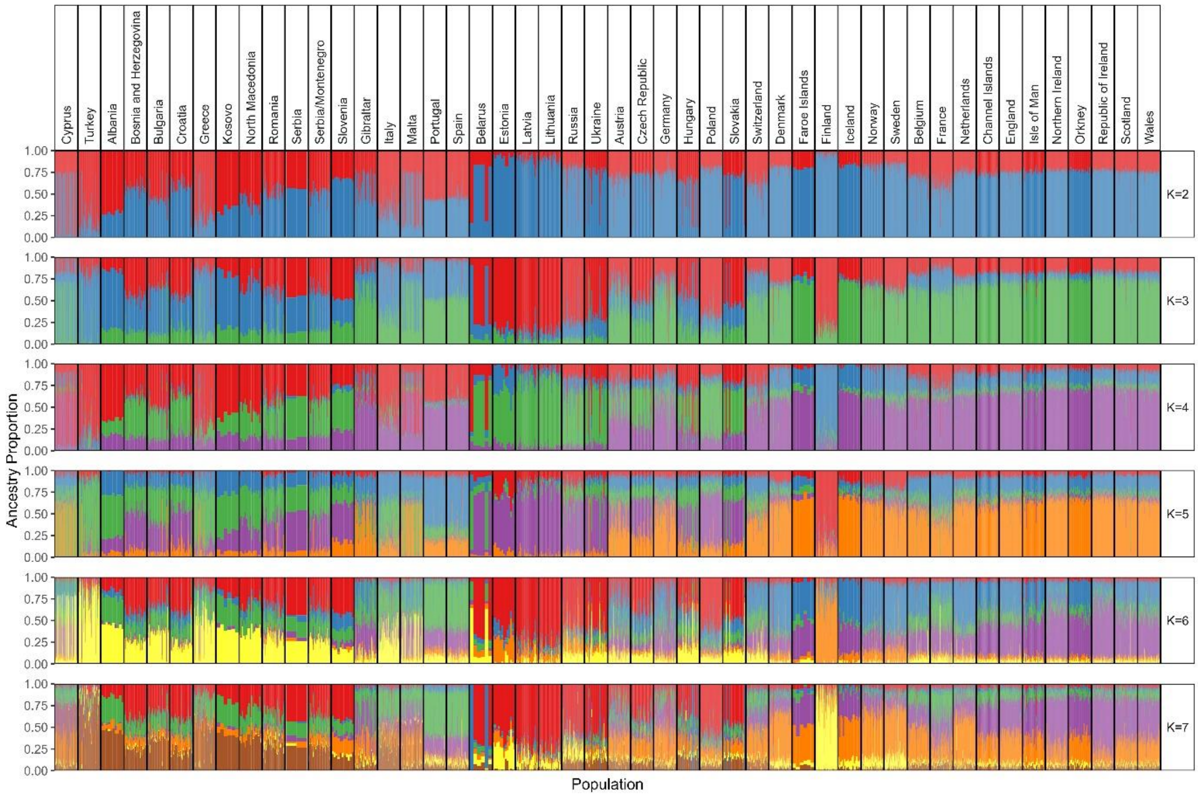

Supplementary Figure 2.10 - ADMIXTURE ancestry components for each sampled European country/region of birth over *k* values 2 to 7. Populations are ordered into the same groups of geographically adjacent regions as in Supplementary Figures 2.1-8.

Supplementary Data 2.3 - Comparison to Human Origins References

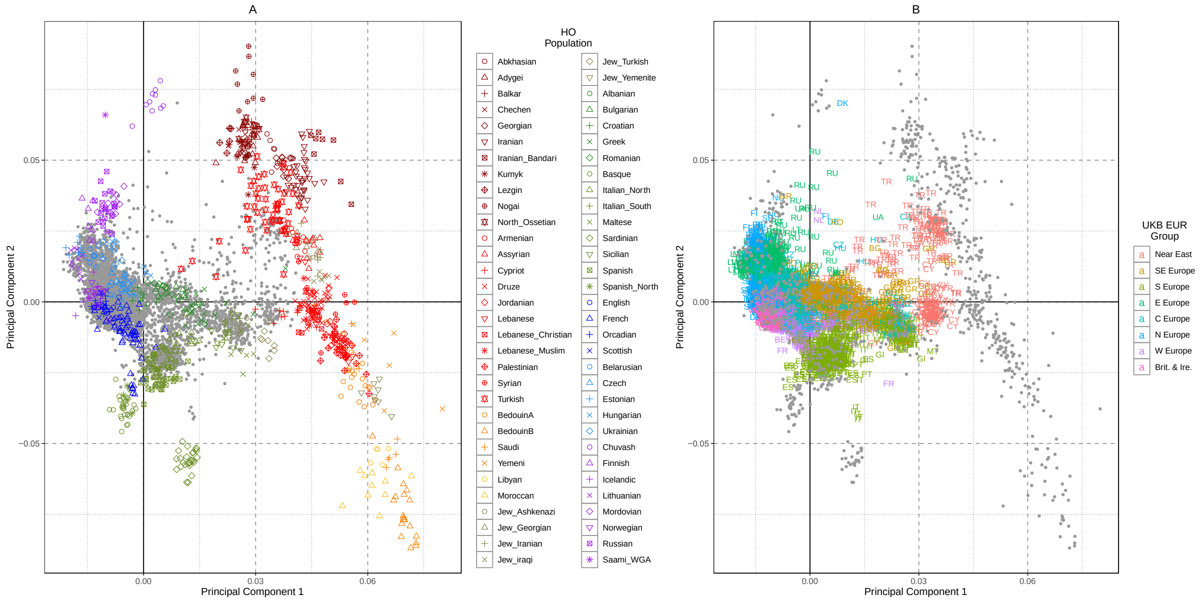

Supplementary Figure 2.11 – Comparison to 5,500 UK Biobank[1] European individuals to 905 West Eurasians from the Human Origins[2] dataset with principal component analysis. UK Biobank individuals were projected onto the genetic variation of the Human Origin references using PLINK[4,5] (see Methods). (A) The principal component (PC) coordinates of the Human origins reference individuals for PC1 and PC2, with label shown by point colour and shape coding, and UK Biobank plotted as grey points. (B) The PC coordinates of the UK Biobank individuals, colour coded according to European meta-region and lettering to show country of birth.

Supplementary Data 3 – Comparison on Linked and Unlinked Methods

Multiple authors have noted the increased power to detect fine-scale genetic structure in populations when utilising "linked" methods (as discussed by Lawson et al[7], Leslie et al[8], and others using European populations[9-12]). To elaborate, "linked" methods are methods which consider the linkage (in the linkage disequilibrium sense) between markers and do not assume that genetic markers are independent with respect to linkage disequilibrium (LD) – i.e., so-called "unlinked" methods.

Indeed, we observe a similar effect comparing the principal components of an unlinked PCA[13] from PLINK[4,5] (**Figure 1**) and principal components calculated from a co-ancestry matrix from *pbwt paint*[14] (**Figure 2**), our equivalent of a linked analysis. Genetic regions of Europe appear to separate out more in this *pbwt*-based PCA than compared to the PCA calculated from unlinked allele-frequency data. As mentioned in the main text Finland appears to be more strongly differentiated in PCA, possibly as a result of the increased haplotype sharing within that population (see Figures 3 and 5). Other populations such as Malta, Turkey and Cyprus, and differentiation between central mainland Europe and Scandinavia to the north appear greater. We attribute this to PCA decomposition of the co-ancestry or genetic-relationship matrices detecting differing relationships along different time frames as haplotype information would be expected to "up-weight" more recent relationships. To further explore this we perform additional analysis, comparing unlinked and linked PCA methodologies on to a well described dataset of European ancestry.

This dataset, the POPRES dataset[15] was used previously by Novembre and colleagues[16] using unlinked methods to strikingly describe the overall European genetic landscape. We investigate whether our results from *pbwt*-PCA are consistent with previous characterisations we analysed the POPRES dataset[15] utilised by Novembre et al[16]. We compared an unlinked method, performing PCA on a *PLINK* generated genetic-relationship-matrix (GRM), to two linked methods - performing PCA on the co-ancestry chunkcounts matrices estimated by *pbwt* and *CHROMOPAINTER*[7].

We selected individuals of European ancestry from versions 1 and 2 of the Population Reference Sample (POPRES) dataset[15] (n=5,917), a DNA resource from multiple studies across the world. We filtered individuals based on the observed country of birth data of their grandparents, selecting individuals whose grandparents were from the same European country. Due to the dataset containing >1000 Swiss samples, we randomly down sampled this label, choosing 100 individuals who were Swiss-French.

Using *PLINK* (ver. 1.9)[4,5], we excluded any strand-ambiguous SNPs (A/T, G/C) and kept autosomal SNPs. Further, we removed SNPs with minor allele frequency (MAF) <2%, missingness >5%, and a Hardy-Weinberg Equilibrium (HWE) p-value <1e-6. Additionally, we removed individuals with SNP missingness >5%. Pairs of related individuals (up to 3rd degree relations) were identified using *KING* (ver. 2.2)[17] and one random individual from the pair was removed. To calculate an unlinked PCA, we utilised a set of SNPs pruned with respect to linkage disequilibrium using the PLINK command --indep-pairwise 1000 50 0.2, performing PCA using the --pca *PLINK* command on these samples and pruned markers ($n_{samples}$ = 954, $n_{markers}$= 103,925).

We divided the unpruned dataset by chromosome and converted these subsets to VCF file format using *PLINK* (ver. 1.9). These genotypes were then phased using *SHAPEIT* (ver. 4.2.1)[18] with effective population size set to 11,418 and using genome map build GRCh37. The phased genotype VCF files

were converted to the hap/sample format using *bcftools* (ver. 1.4.1)[19] which were subsequently painted using (a), *CHROMOPAINTER*, which is a part of the *fs* (ver. 4.1.1) toolset[7], and (b) *pbwt paint* from the pbwt package[14].

To generate the co-ancestry matrixes and perform PCA we carried out the following pipelines. For CHROMOPAINTER, we converted the files hap/sample file set to the *CHROMOPAINTER* input format using scripts provided by the authors of the *fs* utility. Next, the inferred mutation rate and effective population size parameters of *CHROMOPAINTER* Hidden Markov Model were estimated on the autosomal chromosomes (fs "stage 1"). Haplotype sharing in the full dataset was then estimated with these parameters (fs "stage 2") generating co-ancestry matrices of "chunk counts" and "chunk lengths", using default parameters. We calculated principal components on the co-ancestry matrix generated by *CHROMOPAINTER* using R scripts provided by the authors of *fs*. Alternatively, for *pbwt*, we used the phased VCF files to generate a co-ancestry matrix using the *pbwt -paint* command. We calculated principal components on the chunkcounts output from the analysis using the same scripts to perform the *CHROMOPAINTER* PCA, setting the diagonals of the *pbwt paint* co-ancestry matrix to 0. Additionally, we split the countries in the dataset into ten regional labels depending on their geographic position within Europe (see below).

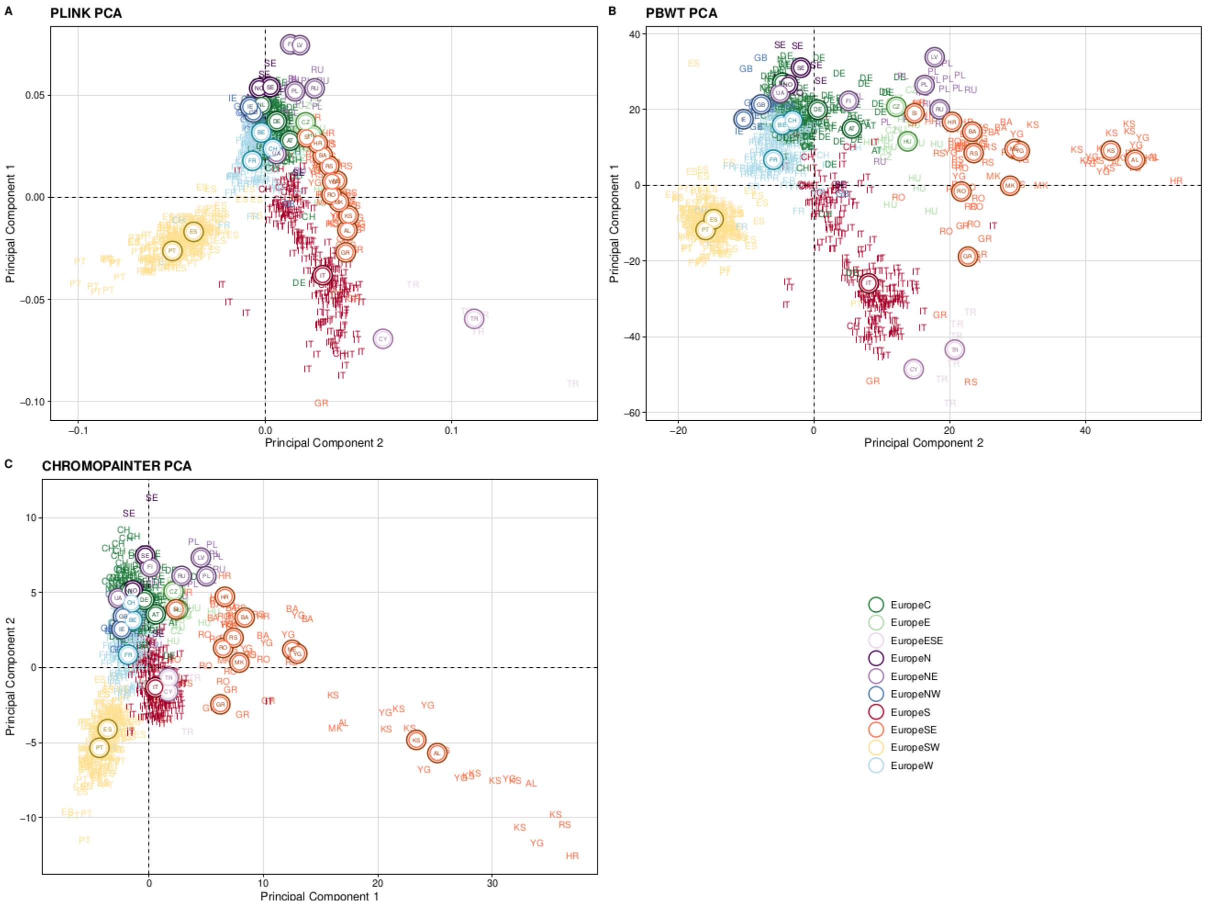

Supplementary Figure 3.1 - Principal component analysis[13] of 954 Europeans selected from the POPRES[15] dataset, calculated from genetic relationship matrix output from *PLINK*[4,5] (A), *pbwt*[14] co-ancestry chunkcounts matrix (B), and *CHROMOPAINTER*[7] co-ancestry chunkcounts matrix. Individual genotypes are shown by letters which encode the alpha-2 ISO 3166 international standard codes, and

are colour coded according to geographic region. The median position for each country label is shown as a label encapsulated by a circle.

In our analysis of comparing linked versus unlinked analyses, we show that European individuals do indeed exhibit great overall separation in principal component space (panel A versus B or C in Figure S3.1). All three PCAs show the defined north to south, and east to west gradients in European genetics[16,20,21], and agree with our own findings using an expanded sample of European genetics (Figure 1). Our results from *pbwt* and *CHROMOPAINTER* show greater separation in south-east Europe, particularly individuals with Kosovan or Yugoslavian grandparental birthplaces. As these analyses are haplotype based this may be due to the relative enrichment of haplotype sharing in this region compared to other populations sampled in the POPRES dataset, consistent with our findings of individuals born from that European region (Figure 4).

Unfortunately, we were unable to analyse the genotypes of the exact individuals reported by Novembre et al[16], leading to a reduction of sample sizes notably from the UK. This may explain that whilst we capture the general structure reported by Novembre et al, there are some topographic differences compared to our Figure S3.1A.

Our comparative results show that each method captures the broad genetic structure in the same dataset, though it appears exactly relationships are weighted differently, hence the differing topologies in PC space. Ultimately, all are informative, though linked methods were able to separate out sub-regions within the same dataset better than unlinked methods in both our analysis of the UK Biobank[1] and POPRES[15] datasets. These results are in agreement with the initial report of the *CHROMOPAINTER* method[7], and findings from groups applying these methods to specific populations[8,22]. This performance is thought as a reflection of haplotypes to better capture information about more recent relatedness between individuals. Therefore, in this analysis of the POPRES dataset, and the comparison between unlinked and linked methodologies, we are confident both are sample capture the major genetic structure within Europe, and our findings from eigen decomposition of the *pbwt paint* co-ancestry matrix is consistent with previous efforts in Europe[16] and findings from using the *CHROMOPAINTER* method[7,8], which unfortunately doesn't scale to this sample size well.

Supplementary Data 4 - Genetic Structure of European Leiden Clusters

Investigating the population structure captured in our sampled of UK Biobank[1] participants with a European place of birth, we performed Leiden[3] clustering of a network of nodes made up of individuals, and edges of the per-individual pair *pbwt paint*[14] co-ancestry estimate (see Figure 2, Methods). To further characterise these clusters, we perform several additional analyses, shown below. The ADMIXTURE[6] ancestry component estimates are the same calculated in Supplementary Data 2.3, but individuals are instead grouped by Leiden cluster membership (Supplementary Figure 4.1). We relabelled the principal component coordinates for the 5,500 UK Biobank individuals projected onto the genetic variation of west Eurasians from the Human Origins dataset[2], labelling the UK Biobank individuals by Leiden cluster (Supplementary Figure 4.2).

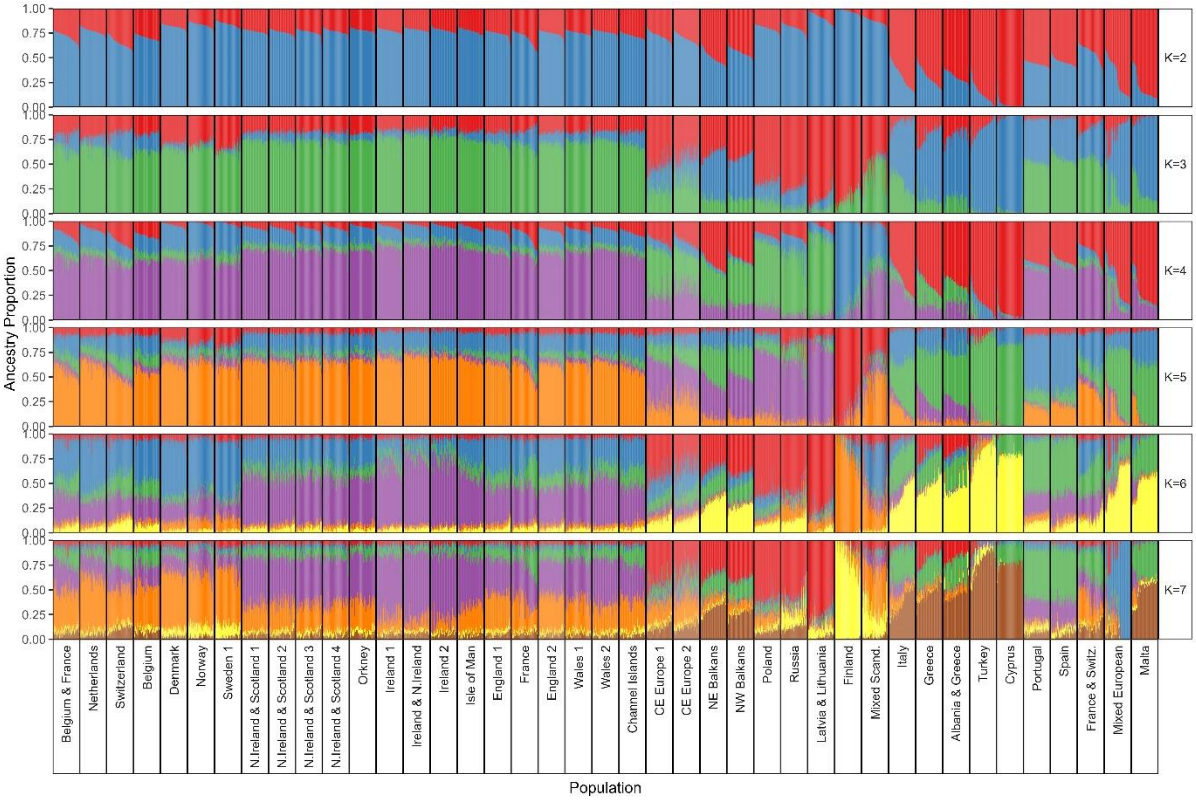

Supplementary Figure 4.1 - ADMIXTURE[6] ancestry component estimates of 41 Leiden[3] clusters of 5,500 UK Biobank[1] participants over *k* values of two through to seven.

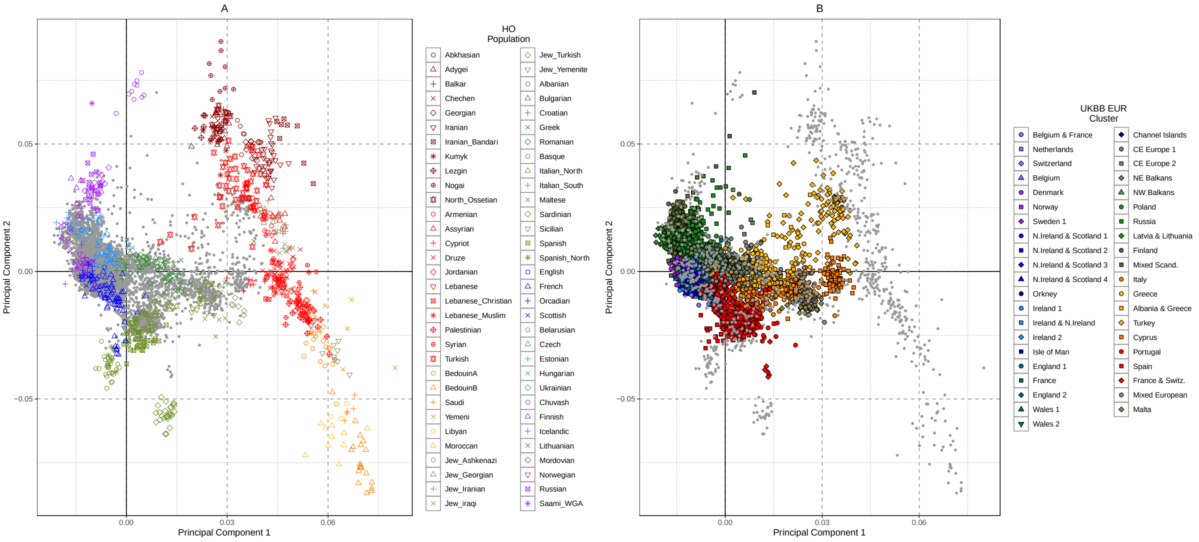

Supplementary Figure 4.2 – Principal component analysis of 5,500 UK Biobank participants with European birth places, projecting onto West Eurasian references from the Human Origins dataset. (A) Each coloured point represents the genotype of one Human Origins reference individual, with UK Biobank individuals represented by grey points. (B). Each colour point represents the genotype of one UK Biobank individual, with Human Origin references shown as grey points.

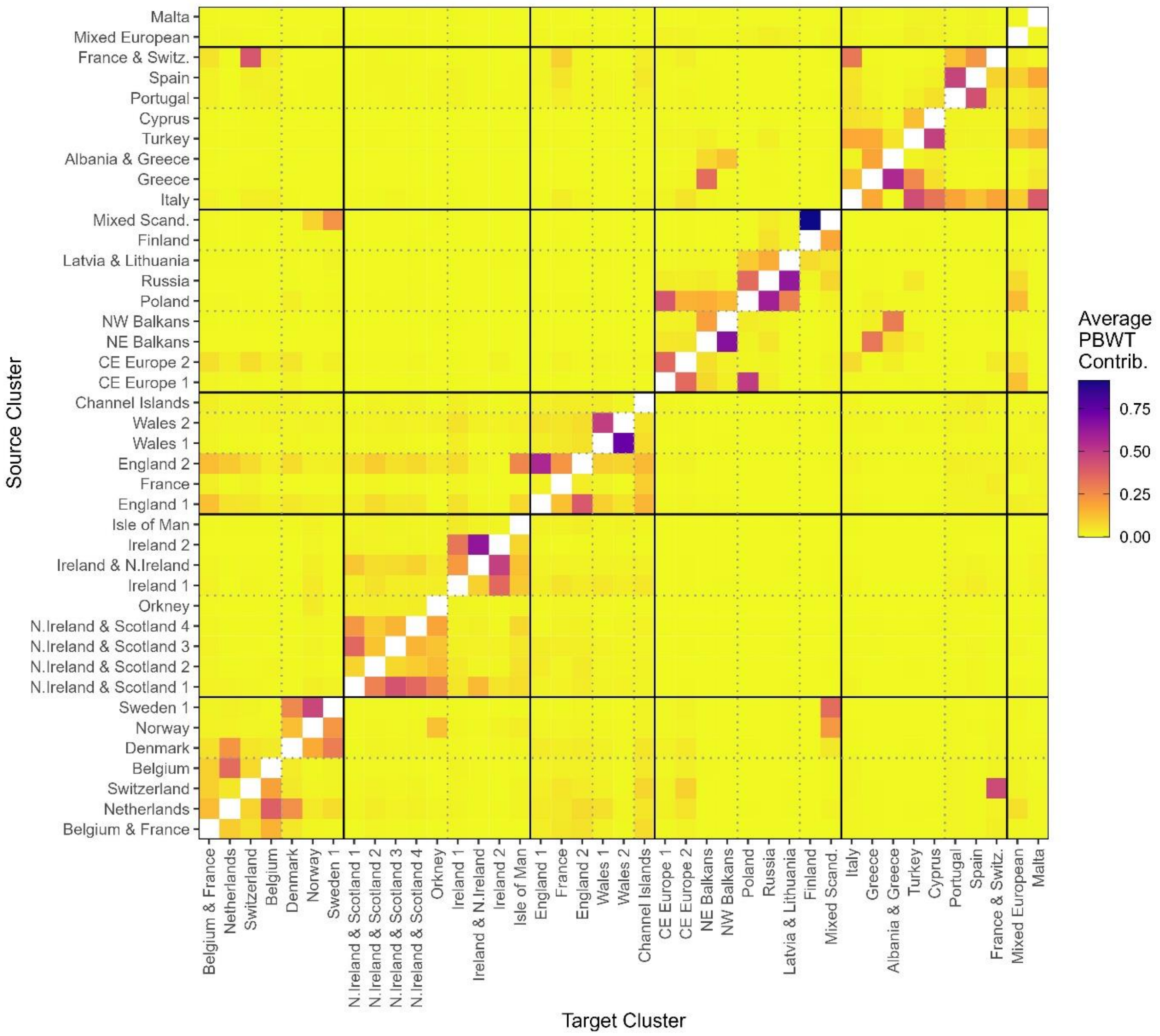

Supplementary Figure 4.3 - Heatmap of average per-cluster pair of haplotype copying profiles between every Leiden cluster as a "target", using every other Leiden cluster as a potential "source" cluster. Average contribution was estimated using an adaptation of a *nnls*-based method[8], using *pbwt paint* co-ancestry estimates as copy vectors.

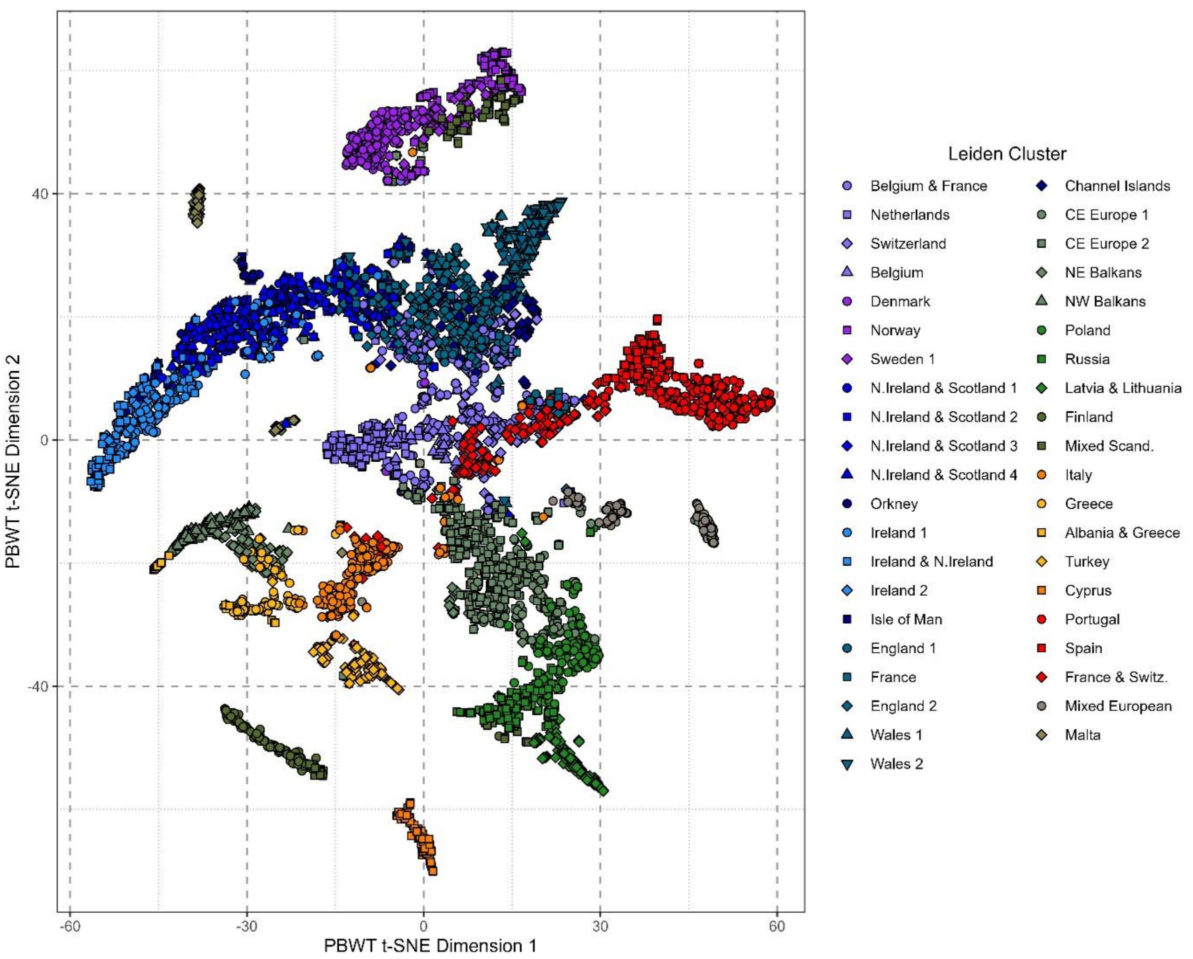

Supplementary Figure 4.4 - Plot of t-SNE[23] decomposition of the top 10 principal components calculated from the *pbwt paint* chunkcounts co-ancestry matrix. t-SNE analysis summarised haplotypic relationships between 5,500 UK Biobank individuals with a European birthplace into two dimensions, and individuals are colour and shape coded according to Leiden cluster membership.

Supplementary Data 5 – Detailed European Genetic Landscape

For the purposes of brevity, in the main manuscript we highlight the primary results from each geographic region sampled from the UK Biobank (UKBB). In this supplementary discussion we describe in greater detail the results of each geographic region.

North-Western Europe

This group of clusters group individuals whose birthplace includes Scandinavia, the Low Countries, France, Switzerland, and the British Isles and Ireland. Three main branches of clusters are detected, one grouping Scandinavian individuals and other continental Europeans, one of individuals from the eastern British Isles, and one of Scotland and Ireland.

Individuals from Scotland and Northern Ireland are still grouped together at this level of clustering, agreeing with previous observations of gene flow between Scotland and the north of Ireland[24]. This is further supported in our *nnls* analysis (**SI Appendix Fig. S4.3**) which models the *N.Ireland & Scotland* clusters with a contribution from the *N.Ireland & Ireland* cluster. We continue observations that individuals with an Orcadian birthplace exhibit high haplotype sharing[8,24] (Figure 3), and have a historically low effective population size (Figure 4) – which also agrees with estimates and time of lowest population size from an analysis of Orcadian individuals with extended recent ancestry from those isles[11]. Interestingly, we identify a discrete cluster of individuals (n=41) predominantly from the Isle of Man (Figure 2). These individuals present a similar profile of modest isolation as Orcadian individuals, with a reduced population size from 30 to 10 generations (Figure 4), modest increase in length of IBD segments (Figure 3), and elevated levels of ROH (Figure 5) - though the latter is not as elevated as Orkney. Previous analysis of genotypes from the Isle of Man agrees with genetic structure[24], though we estimate elevated ROH in this sample compared to that separate of Manx genotypes.

Clusters with English, Welsh, French and Channel Island membership are placed in the eastern British Isles sub-branch. Wales, especially *Wales 2*, shows elevated within-cluster sharing of IBD-segment count compared to other clusters on this sub-branch suggestive of modest isolation. This agrees with previous analyses of samples with extended Welsh ancestry from the Peoples of the British Isles Study[8]. As discussed in the main manuscript, individuals placed within the *Channel Islands* cluster exhibit elevated sharing more of and longer IBD-segments than other Eng. & Wales clusters. These IBD results reflect the sustained lower effective population size than clusters on the same sub-branch, though the *Channel Islands* exhibit evidence of a more modest population contraction than *Orkney*. The 117 individuals with a Channel Island birthplace are not just placed in this cluster of 83 individuals, but also within English (n=11), Welsh (n=4), French (n=5), or Irish/Scottish (n=19) clusters. Compared to Orkney, whose cluster shows equivalent IBD sharing to *Channel Islands*, individuals with a Channel Islands birthplace are more distributed in other clusters – suggesting that whilst there may be an island population of interest to haplotype mapping, the islands are less isolated which reflects their geographic position in-between France and England.

Within the continental WE branch of clusters, Belgian, French, and German individuals are clustered together (*Belgium & France*) and are grouped with clusters of separately Swiss (*Switzerland*) and Dutch (*Netherlands*) predominant membership. A smaller cluster, *Belgium* (n=53), is detected. This cluster does not appear to present high haplotype sharing or evidence of population contraction, and in *nnls* analysis shares a haplotype profile predominantly donated from *Netherlands*. Further profiling of this cluster is difficult, though there appears to be a genetic continuity between Belgium and Netherlands, which is consistent with their geographic proximity and lack of large-scale geographic barriers.

Genetically, Swiss individuals project between France, Germany, and Italy in PCA, reflecting their geographic location. We estimate *Switzerland* to have a recent effective population size to be equivalent to *Belgium* or the *Netherlands*, but no evidence of substantial isolation.

Within the Scandinavian sub-branch, we detect three clusters which group individuals from Denmark, Norway, and Sweden. These individuals project in-between northern continental Europe and Finnish individuals, showing within-cluster IBD profiles equivalent to north-western Britain or Ireland. Our small sample of Icelandic individuals (n=19) are grouped with Norwegians in *Norway*, and as described in the main manuscript show evidence of isolation consistent with the population history of that island[25]. Five out of six sampled Faroese individuals are grouped into the *Sweden* cluster. Similarly to the Icelandic individuals in *Norway*, these Faroe Islanders show substantially higher IBD-segment sharing than Swedish individuals placed in the same cluster. The average pair of Faroe-Faroe individuals share 90 cM of IBD segments > 1cM, sharing 28 segments each of which are on average 3.2 cM long. The average Swedish-Swedish individual pair share a total of 21 cM over 14 segments, each of which are 1.5 cM long. The low sample size of the Faroe Islands means it is unclear just how representative these results are, though a recent study Faroese genotypes indicates the population does indeed exhibit unsurprising hallmarks of isolation[26] – though we are unable to extend and refine this picture as has been possible in Malta.

Central-Eastern Europe

The second branch of Leiden clusters groups individuals with a birthplace from the centre and east of Europe, from Germany in the west to Russia in the east. This branch contains several sub-branches which group individuals from geographically adjacent locations in Europe; NE Europe (with Baltic, Polish, and Russian membership), CE Europe (with membership from the north of the Balkans and the centre and east of Europe), and Finland as an outgroup.

Within the CE European group of clusters there are two sub-groups, each with two clusters. The first, *CE Europe 1* and *2* cluster individuals predominantly from Germany, Austria, Poland, the Czech Republic, and Hungary. *CE Europe 1* appears to group individuals of a more easterly birthplace, and in *nnls* analysis its haplotype profile includes more of a contribution from the *Poland* cluster, whereas *CE Europe 2* contains more individuals from Austria and Germany and a slightly higher contribution from *Switzerland* in *nnls* analysis. In analysis of within-cluster haplotype sharing *CE Europe 1* shows modest elevation of IBD-segment sharing (Figure 3) consistent with a slightly lower historical population size, which our N_e estimates from 30 generations ago agree with. Within the north of the Balkans, we observe a general east-west divide in our clustering and PCA results (Figure 2). With IBD-segment sharing as well as ROH information, it appears *NW Balkans* shows modestly elevated levels of haplotype sharing consistent with a lower effective population size, this is supported by a consistently lower effective population size in *NW Balkans* compared to *NE Balkans*.

In our main manuscript we discuss the results of the Finnish, E Europe, and northern Balkans clusters in greater detail, noting that our results in Finland agree with existing literature on this well studied population. In NE Europe the UK Biobank sample largely agrees with the Human Origins references, though our Russian samples more project towards Baltic/Ukrainian genetic space as opposed to the Human Origins Russians, who project more towards Chuvash or Saami – though there is overlap in the UKBB and Human Origins Russians. We suspect that the UKBB sample of Russian ancestry is therefore biased towards European ancestry rather than the central Eurasian or Caucus/related ancestry that is represented by the Chuvash or Saami in the Human Origins West Eurasian sample. This heterogeneity in sampled Russian ancestry can be explained by the variation in sampled ancestries within the Russian Federation, with ancestry in samples closer to Europe exhibiting more European-modal ancestry[27].

Southern Europe

The third and final group of Leiden clusters contains individuals with a birth-place from regions within or north of the Mediterranean. The major sub-groups include the islands of Malta and Cyprus (grouped for convenience, but genetically distinct), Italy, the Iberian Peninsula, SE Europe – which includes Greece and Turkey, and a cluster of individuals with a heterogenous mixture of birthplaces (*Mixed European*). We discuss the full results of the large *Malta* cluster in our main manuscript – but briefly our results show that Malta is genetically distinct and presents evidence of genetic isolation with high haplotype sharing and genetic distances to non-Maltese clusters. In PCA with UKBB individuals, or projected onto Human Origins genetic variation, Maltese individuals project close with *Cyprus 2* and *Italy 2*.

Geographically neighbouring Malta, individuals from Italy are grouped into one Leiden cluster, though PCA divides individuals into predominantly two clusters, which appear to reflect the general north-south divide previously detected in Italian genetics[10]. *Italy* haplotype contributions come from the mixed *France & Switz.* cluster, as well as *Turkey* and Greece - which agrees with its position between western Europe and the south-east of Europe and the Near East[10]. This is associated with a consistently high historical effective population size compared to other European regions (Figure 4).

Within Iberia, the predominant divide is between Spanish and Portuguese samples, in agreement with recent analyses[12]. *Spain* and *Portugal* show evidence of reciprocal admixture in *nnls* analysis. *Portugal* appears to have modestly elevated haplotype sharing compared to *Spain*, consistent with its geographic position at the end of the Iberian Peninsula. This haplotype sharing is observed in both IBD and ROH segment sharing, where elevation of the latter appears to be driven by predominantly longer ROH than greater numbers of ROH (Figure 5). As discussed in the main manuscript we detect small group of Spanish individuals within *Spain* which is genetically distinct from other Spanish clusters and exhibits elevated IBD-segment sharing. With evidence from co-analysis of references from the Human Origins dataset, we suspect that this cluster represents ancestry from the north-east of Spain and the Basque population. Further sample annotation is not available for these samples, so we are unable to definitively prove this.

Within SE Europe, we cluster individuals from Albania, Greece, North Macedonia, Turkey. Italian clusters from an outgroup to this sub-branch along with *Cyprus*. We observe some signal of haplotype sharing between *Italy* and *Greece*, as well as sharing between *Italy* and *Turkey*. As discussed in the main manuscript, we detect a small cluster of Greek, Albanian, and North Macedonian membership, *Albania & Greece*. It is unclear if this small cluster is largely represented of this geographic region, though there is no obvious reason that the UKBB would have sampled a specific community from this area of Europe with history of isolation. *Turkey* and *Cyprus* exhibit evidence of isolation that is consistent to some degree of consanguinity in inbreeding coefficient analysis. In comparison to Turkish references from the Human Origins dataset *Turkey* co-segregates with other Turkish references – forming a tight cluster in PCA in the centre of the distribution of Turkish genotypes. We see a similar relationship between *Cyprus* and Cypriot Human Origins references. This appears to be a relatively isolated Turkish community, captured within the UK Biobank. Lastly, as further expanded upon in the main manuscript as well as in **SI Appendix Data 7** we detect a connected community of individuals with a mixture of birthplaces across Europe. In analysis of ancestry, population structure, and characterisation of demographic history we propose this *Mixed European* cluster represents a community of Ashkenazi Jewish participants in the UK Biobank. For more information and analysis, see **SI Appendix Data 7**.

Supplementary Data 6 - Malta

In analysis of European ancestry sampled within the UK Biobank, we report to our knowledge the largest dataset of Maltese genotypes publicly available. As part of the sampling scheme, we randomly down-sampled individuals from countries/regions of birth which had more than 200 individuals to a randomly selected 200 individuals. This was to both reduce sample size and to control for uneven sampling rates across European regions, for example there were over 1000 individuals with a German place-of-birth compared to 180 Swiss. We randomly sampled 200 Maltese individuals out of a possible 362.

Investigating the population structure in this sample of European genotypes we observe population structure within our Maltese sample (see **Figure 1**, and **Figure S2.2**), where three broad clusters emerge in principal component space. Further investigating this, we find that principal component (PC) seven of the PLINK[4,5] PCA, of which PC one and two are shown in Figure 1, also separates these individuals, see **Figure S2.2**. When we code the sampled Maltese individuals instead by Leiden cluster membership of the *Malta* cluster or not, the Leiden clustering clearly separates out these two groups, see below:

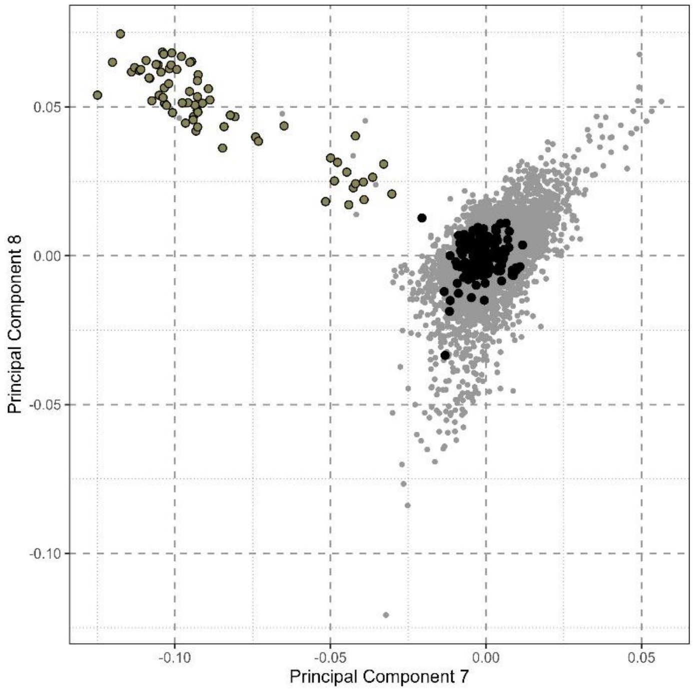

Where the *Malta* cluster is comprised of the Maltese individuals at the terminus of the cline away from other Europeans, as well as the smaller cluster of Maltese individuals which project in-between that group of Maltese and other Europeans. These two groups of Maltese individuals within the *Malta* Leiden cluster also show two patterns of IBD sharing within the cluster (**Figure 3**), with the intermediate Maltese individuals in PC seven also intermediate in within-cluster IBD-sharing estimates.

Supplementary Data 7 - Mixed Europeans

In our analysis of European genotypes sampled in the UK Biobank[1] we have identified a genetically connected community of individuals with birthplaces across Europe, though with a slight bias towards central/eastern European countries of birth. Despite being geographically dispersed, as represented by birthplace information, these individuals project along a single principal component (principal component nine in **Figure S2.9**) as well as are grouped as a single cluster by application of the Leiden algorithm (**Figure 2**). Compared to the rest of the European sample, a core group of these *Mixed European* individuals project towards individuals with a Near East birthplace (**Figure 2**), and project towards Ashkenazi or Turkish Jewish references from the Human Origins[2] dataset. Furthermore, in our *nnls* based analysis (see Methods, **Figure S4.3**), the haplotype sharing of these individuals can be modelled as a mixture of Near Eastern (*Turkey*: 0.12), southern European, (*Italy*: 0.10, *Spain*: 0.10), and central or Eastern European sources (*Poland*: 0.14, *CE European 1*: 0.13, *Russia*: 0.07).

In addition to this genetic structure, individuals within this cluster exhibit elevated within-cluster IBD-segment sharing (**Figure 3**), a historically low effective population size with recent population expansion (**Figure 4**) and elevation of ROH sharing. This is consistent with a small isolate population that has experienced a population size bottleneck around 30 generations ago. These demographic results, with analysis of population structure in the context of Europe and the Middle East is suggestive that this is a community of Ashkenazi Jews. This would be consistent with previous analyses of the UK Biobank which detected such a community[28], as well as the population history of European Ashkenazi Jews as previous modelled in more detail with individuals recruited from that community[29].

To further confirm this, we performed an additional analysis with references from the Human Origins dataset. Using the same methodology to project the UK Biobank individuals onto western Eurasian genetic variation, we selected Human Origin West Eurasian individuals, as well as Yoruban references form that dataset, and merged this genotype data with the UK Biobank dataset, leaving **6,476** individual genotypes of **61,217** common SNPs after the same quality control thresholds as in the projected PC analysis. Analysing a subset of these markers which were in approximate linkage (filtering SNPs with the PLINK[4,5] option --indep-pairwise 1000 50 0.2) we tested allelic sharing using *f*-statistics implemented in the R package ADMIXTOOLS2 (manuscript in prep). For each Leiden cluster we tested allelic sharing between the closest population reference in the Human Origins dataset and Human Origin Ashkenazi Jew references in the form:

$$f_4(\text{Yoruban, Y; Ashkenazi Jew, European Reference})$$

The f_4 estimates for each Leiden cluster are shown below. Along the y-axis is each Leiden cluster tested in "quotation marks" and the paired population reference chosen from the Human Origins dataset. "*Mixed A*" and "*Mixed B*" refer to two sub-groups of the *Mixed European* cluster which are expanded upon below. With the exception of *Malta*, *Turkey*, and *Cyprus* (i.e., the UK Biobank clusters closest to Near Eastern populations in PCA) all European clusters are significantly differentiated from the Human Origin Ashkenazi Jew references. Both *Mixed European* groups, *Mixed A* and *Mixed B* shared significantly more alleles with Ashkenazi Jew than Turkish references as well as sharing more allelic drift with Ashkenazi Jew references than Turkish Jews (see below).

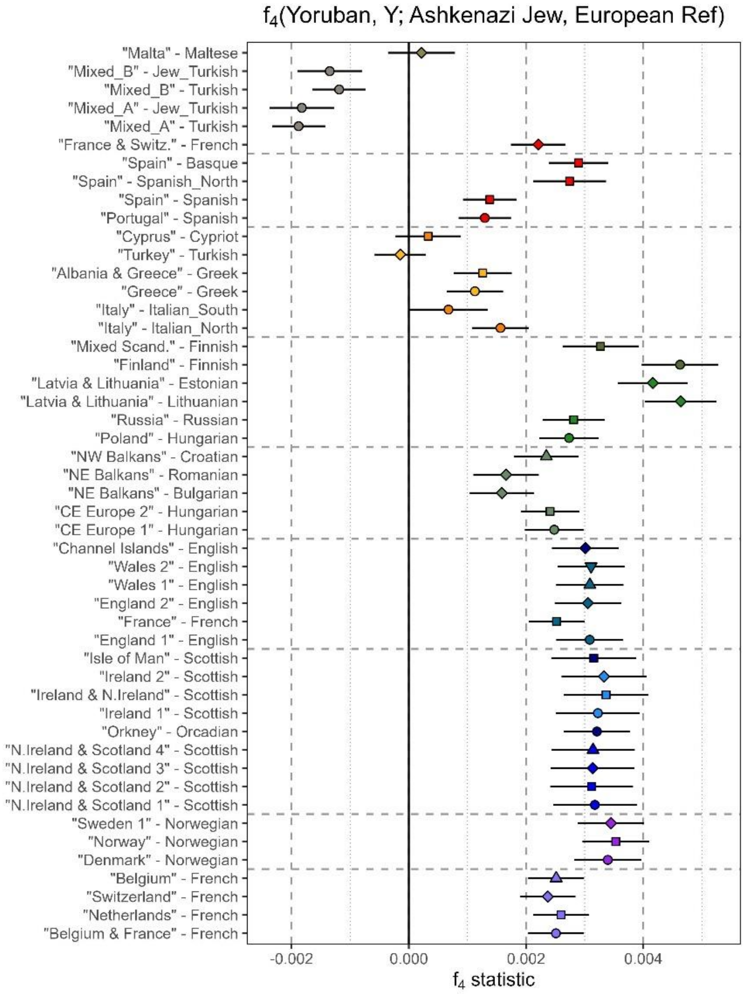

In PCA of the *pbwt paint* co-ancestry matrix, we observed this *Mixed European* cluster separate along PC three, see below. Similarly to individuals placed in the *Malta* cluster, there appears to be two groups of individuals along this PC, which we divided into two groups, *Mixed A* (*Mixed European* individuals to the left of the red line below) and *Mixed B* (*Mixed European* individuals to the right of the red line).

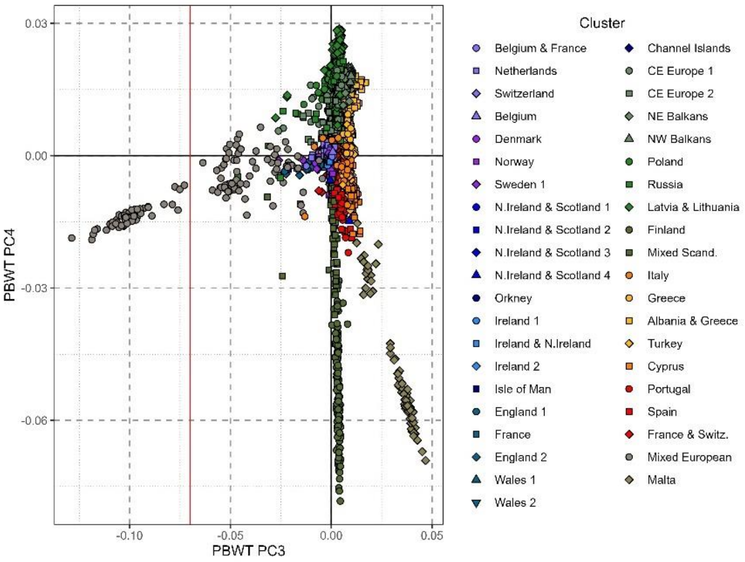

We next tested if these two groups within *Mixed European* presented different haplotype sharing profiles with the rest of our European UK Biobank dataset. We performed a modification of our *nnls* analysis (see Methods), this time modelling each *Mixed A* and *Mixed B* cluster as a mixture of any other Leiden cluster (with the exception of the other *Mixed European* sub-group). We present the results below, finding that the *Mixed B* cluster presents a more heterogenous sharing profile consistent with admixture (which would be consistent with ADMIXTURE estimates (see **SI Appendix Data 4**)).

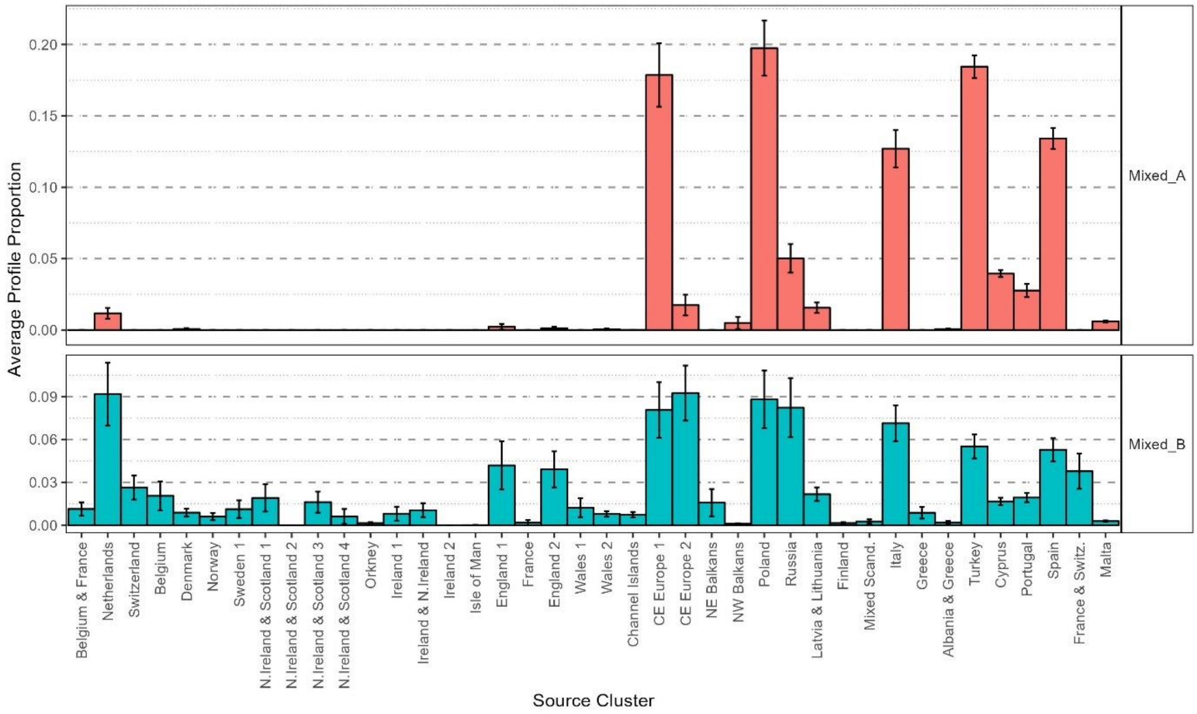

Further testing evidence of *Mixed A* grouping individuals more genetic isolated we recorded levels Runs of Homozygosity (ROH) between the groups, finding that on average individuals placed in *Mixed A* carry more, and longer, ROH than individuals. These differences are significant comparing either the average length of ROH an individual carries between the groups (Mann-Whitney U p-value: <0.0001) or the number of ROH (Mann-Whitney U p-value: <0.0001).

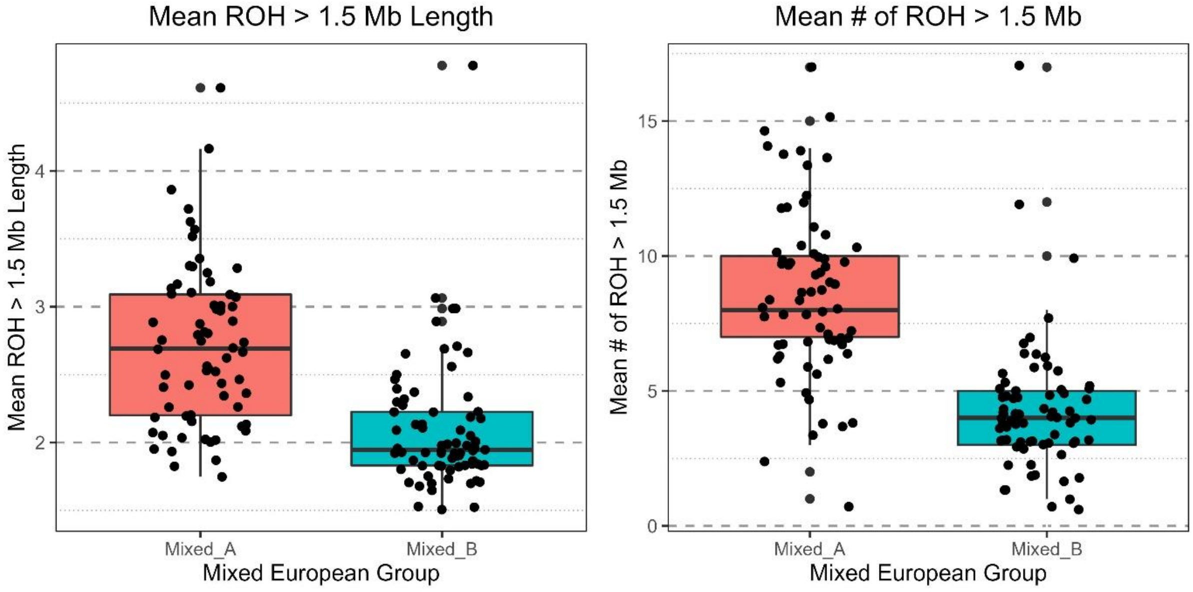

We further explored the degree of haplotype sharing between and within these groups by recording the total length and number of IBD-segments > 3 cM and < 30 cM[29] shared within and between *Mixed A* and *Mixed B* and a comparative cluster, *England 2* using the same methodology as shown in **Figure 3** and described in Methods. We find that IBD segments are slightly longer within and between *Mixed A* and *Mixed B* (below), and that the average *Mixed A* pair of individuals (i.e., individuals both placed in the *Mixed A* cluster) share a number higher number of IBD segments than the average pair of *Mixed B* individuals. Interestingly the average *Mixed A* - *Mixed B* pair of individuals share more IBD segments than the average *Mixed B* pair, suggesting the *Mixed B* are heterogenous within that group.

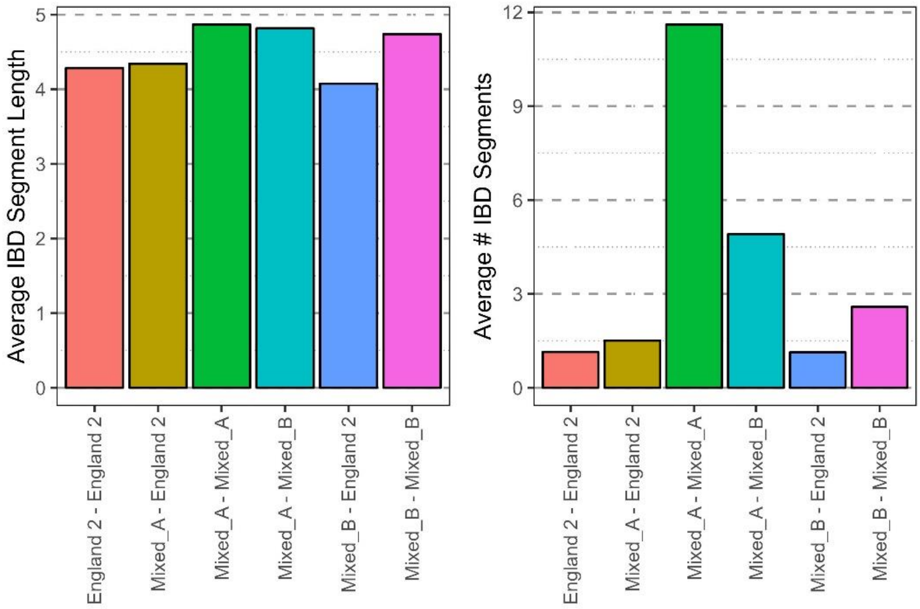

Lastly, we estimated the effective population size of each of the clusters *Mixed A* and *Mixed B* using IBD-segments and IBDNe[30], characterising the haplotype sharing and population structure in terms of historical population size. We find that whilst both present evidence of historical population contraction, we record a much minimal historical population size for *Mixed A* and *Mixed B*, consistent with greater isolation.

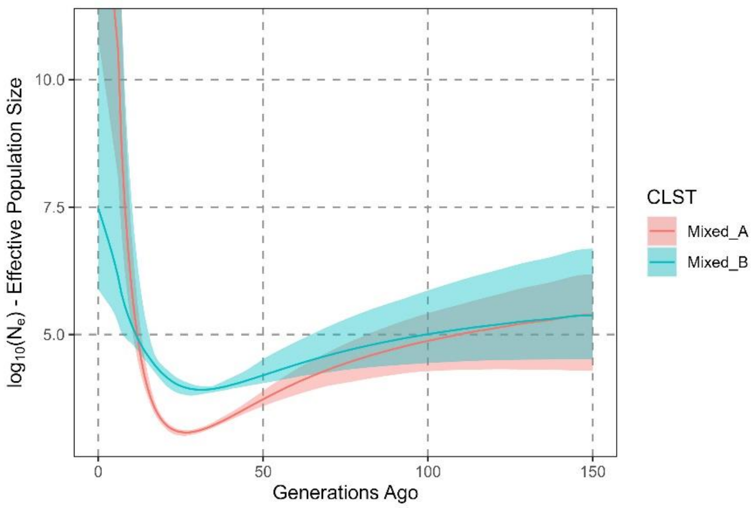

Together these results suggest these two groups represent structure within the sampled community from the UK Biobank, one more isolated than the other. The latter which has experience greater

admixture with European regions, and whose members are less genetically connected (as measure by IBD segment sharing) than the former.

Supplementary Data 8 - IBD Ne Curves

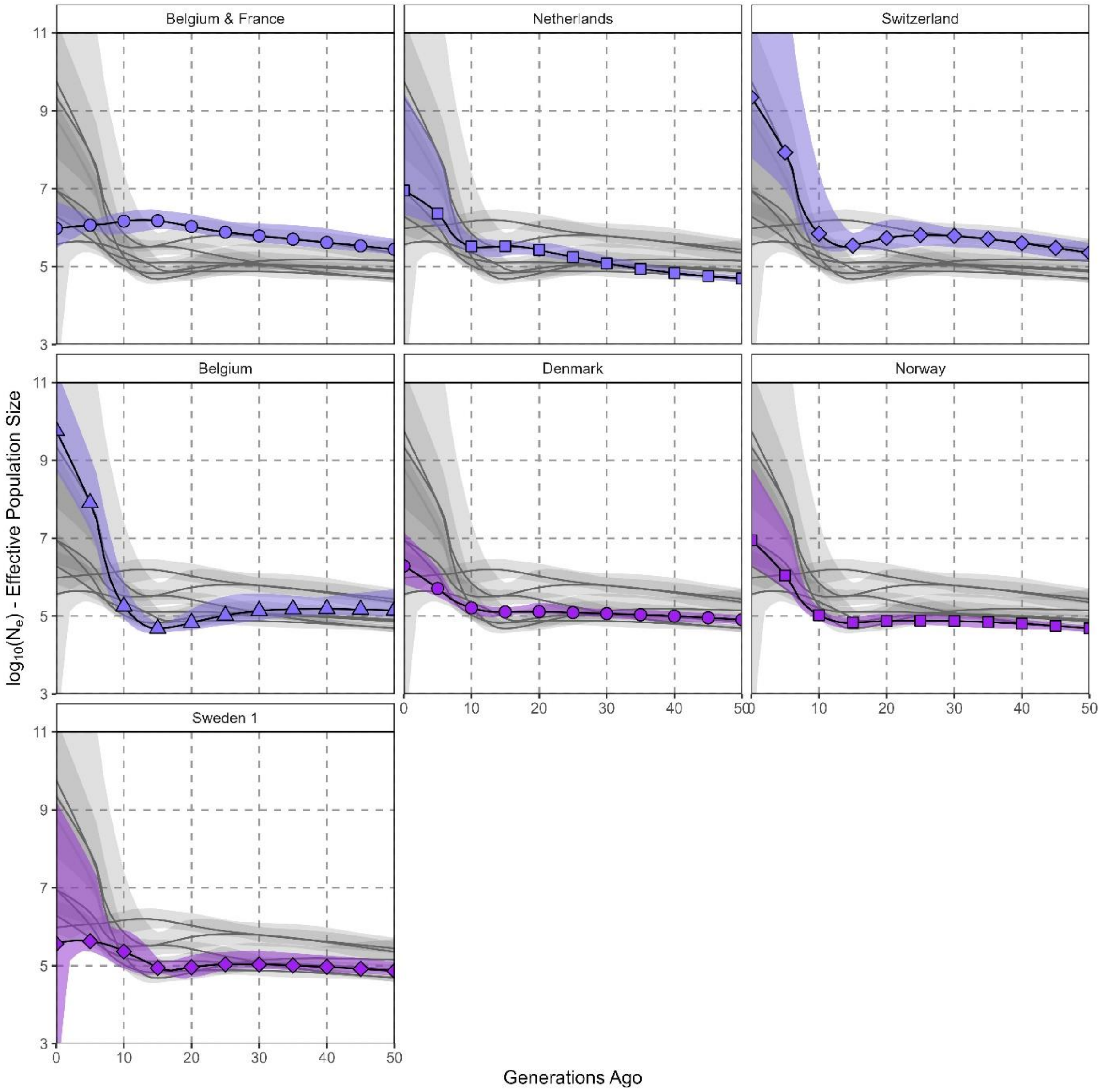

Supplementary Figure 8.1 – Historical effective population size (N_e) of Leiden clusters grouping individuals with a north-western European birthplace. Each panel shows the estimate for one cluster, with grey curves showing the estimates for all other north-west European clusters. Shading indicates the 95% confidence intervals for the estimates.

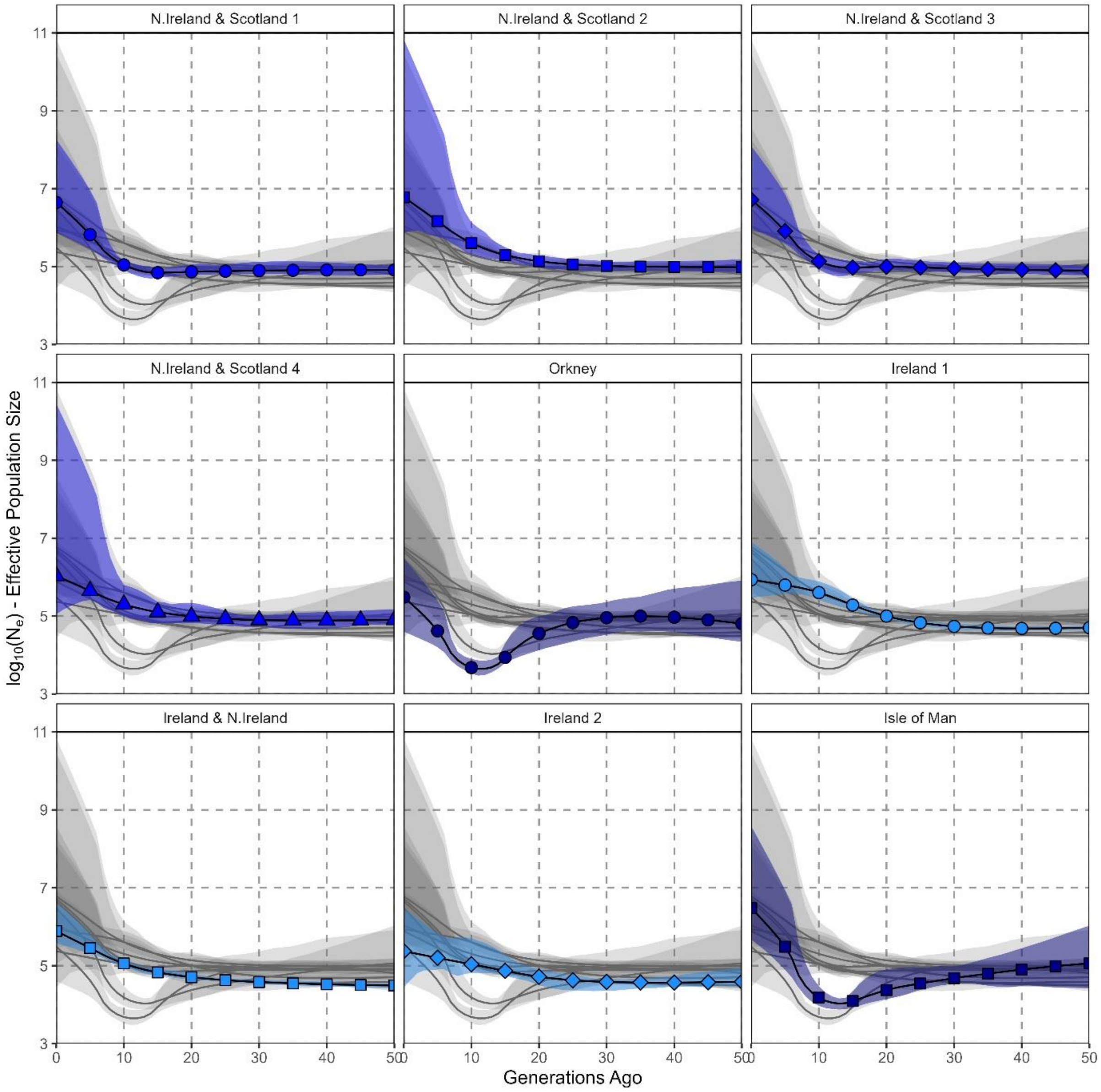

Supplementary Figure 8.2 – Historical effective population size (N_e) of Leiden clusters grouping individuals with a northern Britain or Irish birthplace. Each panel shows the estimate for one cluster, with grey curves showing the estimates for all other northern British or Irish clusters. Shading indicates the 95% confidence intervals for the estimates.

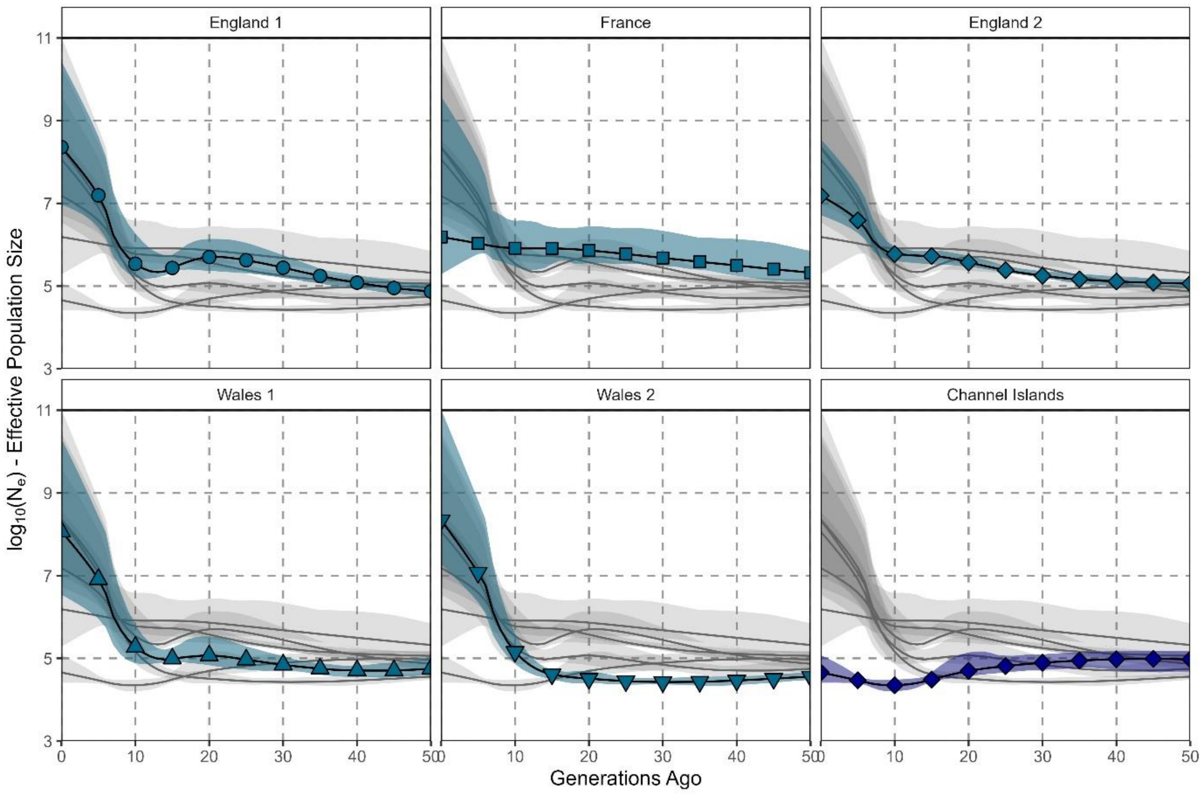

Supplementary Figure 8.3 – Historical effective population size (N_e) of Leiden clusters grouping individuals with an English or Welsh birthplace. Each panel shows the estimate for one cluster, with grey curves showing the estimates for all other southern British clusters. Shading indicates the 95% confidence intervals for the estimates.

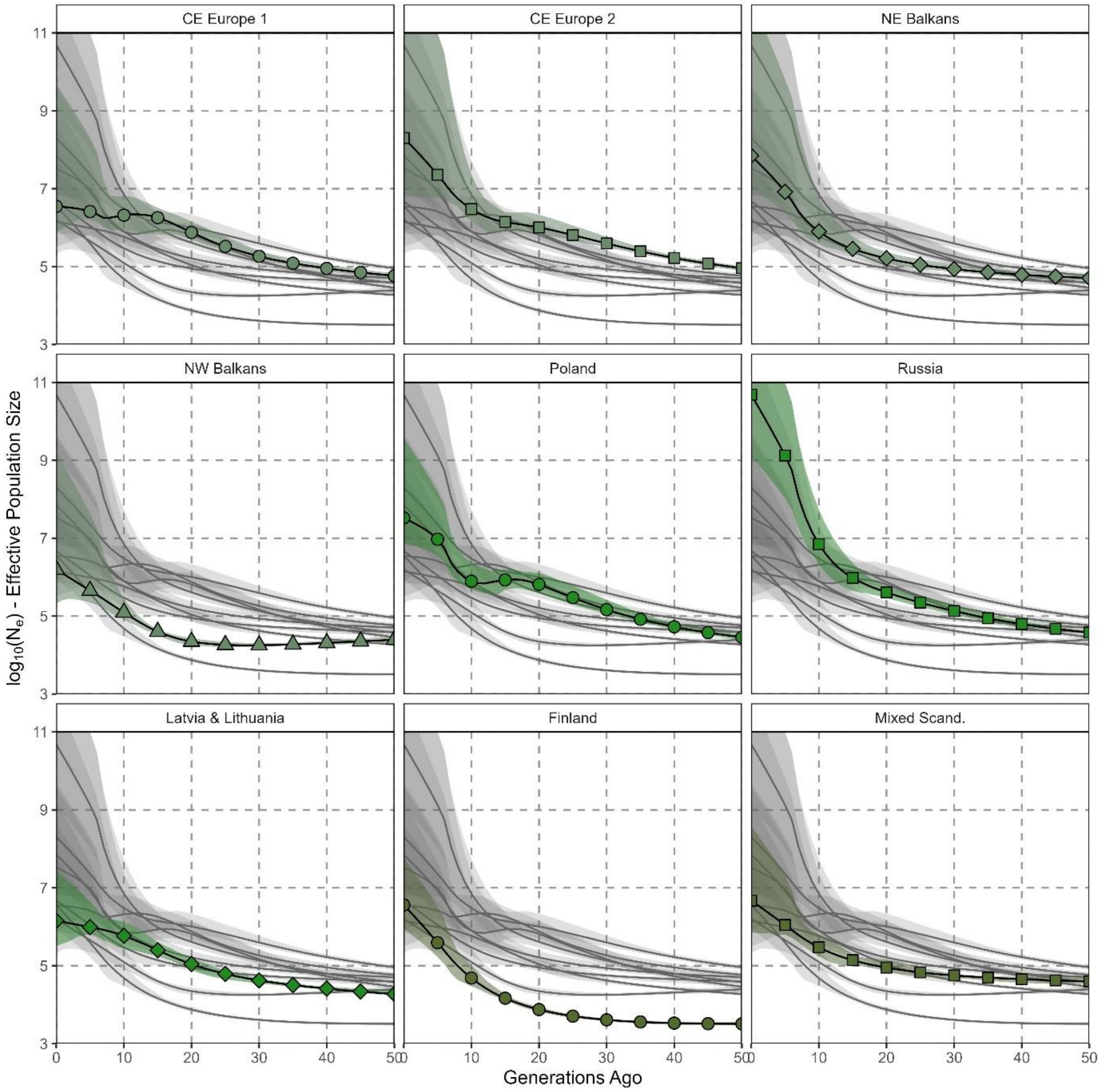

Supplementary Figure 8.4 – Historical effective population size (N_e) of Leiden clusters grouping individuals with a central, northern, or eastern European birthplace. Each panel shows the estimate for one cluster, with grey curves showing the estimates for all other central, northern, or eastern European clusters. Shading indicates the 95% confidence intervals for the estimates.

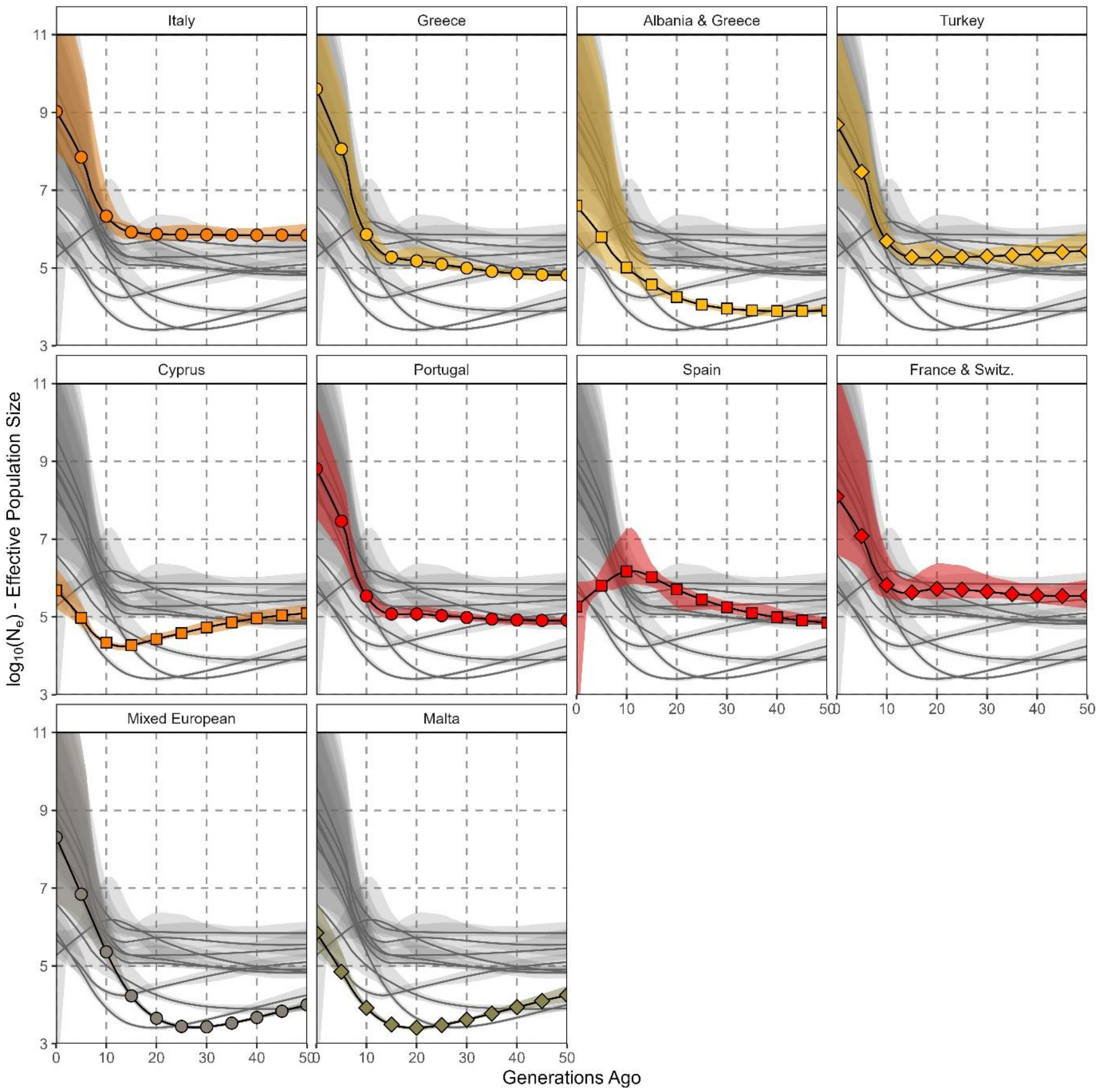

Supplementary Figure 8.5 – Historical effective population size (N_e) of Leiden clusters grouping individuals with a southern European birthplace. Each panel shows the estimate for one cluster, with grey curves showing the estimates for all other south European clusters. Shading indicates the 95% confidence intervals for the estimates.

Supplementary Data 9 - Spain

In analysis of within-cluster Identity by Descent (IBD) segment analysis we detect a number of individuals placed in the *Spain* cluster who present high levels of within-cluster IBD sharing compared to other individuals with a birthplace in Spain (**Figure 3**). In principal component analysis (PCA) of the *pbwt paint* co-ancestry matrix we observe a group of 12 *Spain* individuals who project away from the rest of the cluster on principal component (PC) one, as well as PC six. This is shown below, with the fifth and sixth PCs plotted and individuals labelled by Leiden cluster, and a horizontal black line indicating the cut-off threshold for identifying "*Spain* Outliers" along PC six.

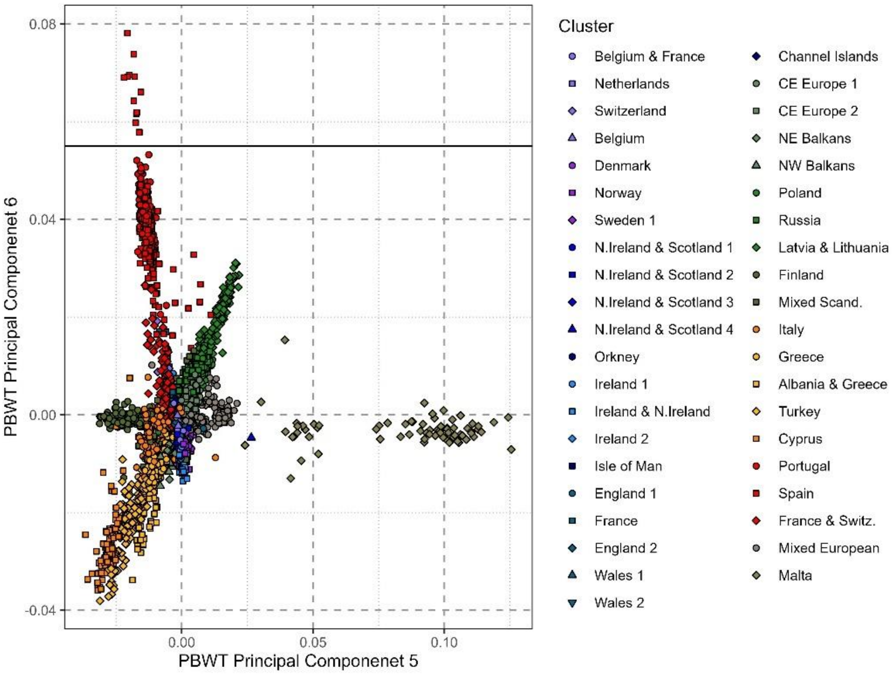

When we plot PCs one and two of the PCA decomposition of the *pbwt paint* co-ancestry matrix, highlighting these 12 individuals, we find that these individuals are the *Spain* outliers along PC one as well, see below. The *Spain* outliers are highlighted in black.

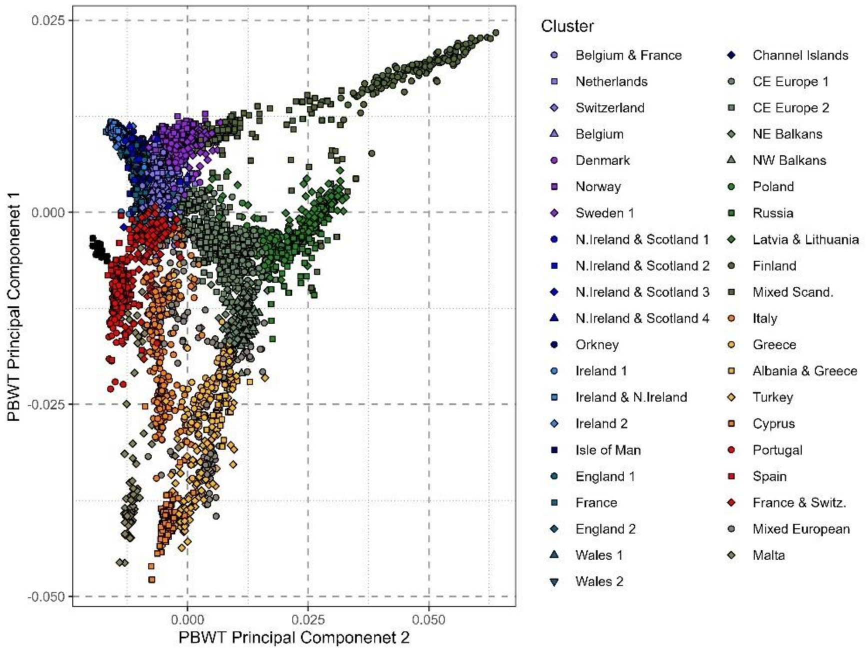

Due to the high levels of IBD segment sharing amongst these 12 individuals (**Figure 3**), we recorded the levels of IBD segment sharing between these *Spain* outlier individuals and the rest of the *Spain* cluster, as well as the levels of sharing between the two Spanish groups. We recorded total length of IBD (below left), and the number of IBD segments (below right), finding that the *Spain* outliers indeed share on average more IBD segments within, as well as between the outliers and the general *Spain* cluster, than the average within *Spain* pair of individuals.

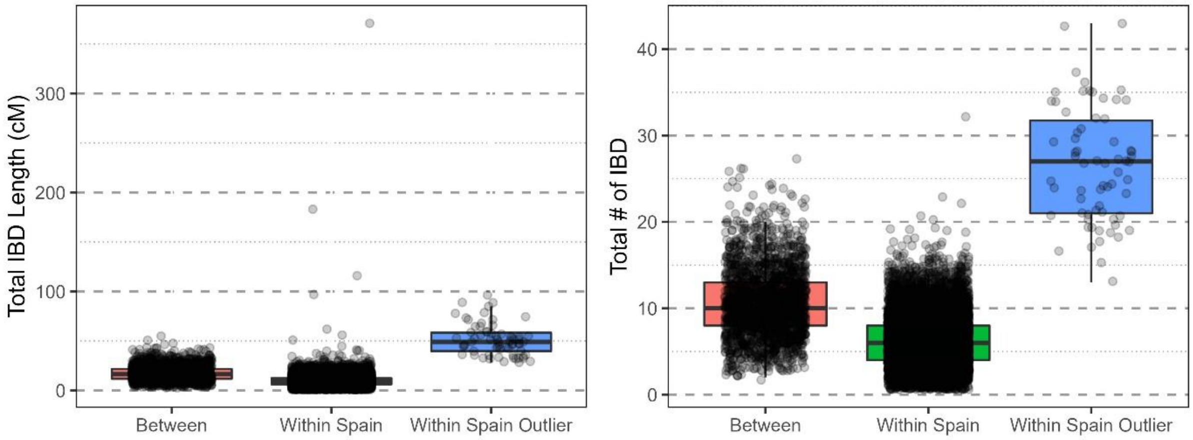

This increase in haplotype sharing is also mirrored in levels of Runs of Homozygosity (ROH), see below, where the average total length of ROH > 1.5 Mb in length is elevated in the *Spain* outliers.

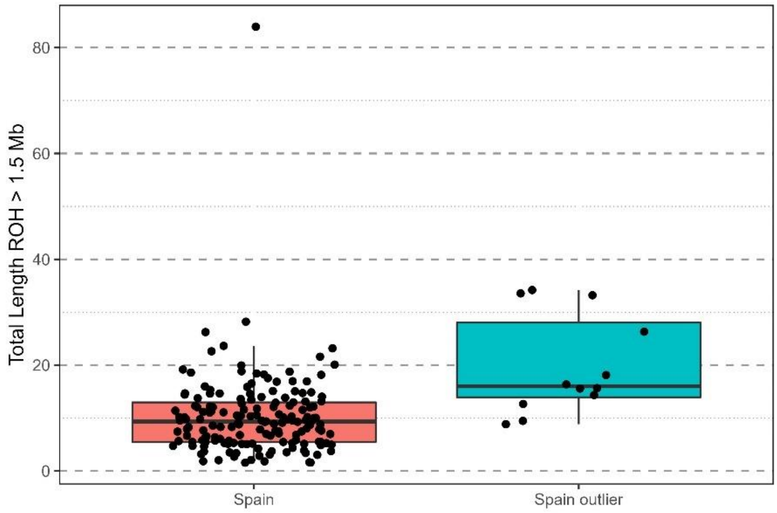

Furthermore, when projected onto the genetic variation of west Eurasian population references from the Human Origins dataset[2] the *Spain* outlier individuals project towards the variation of Basque and northern Spanish references, see below.

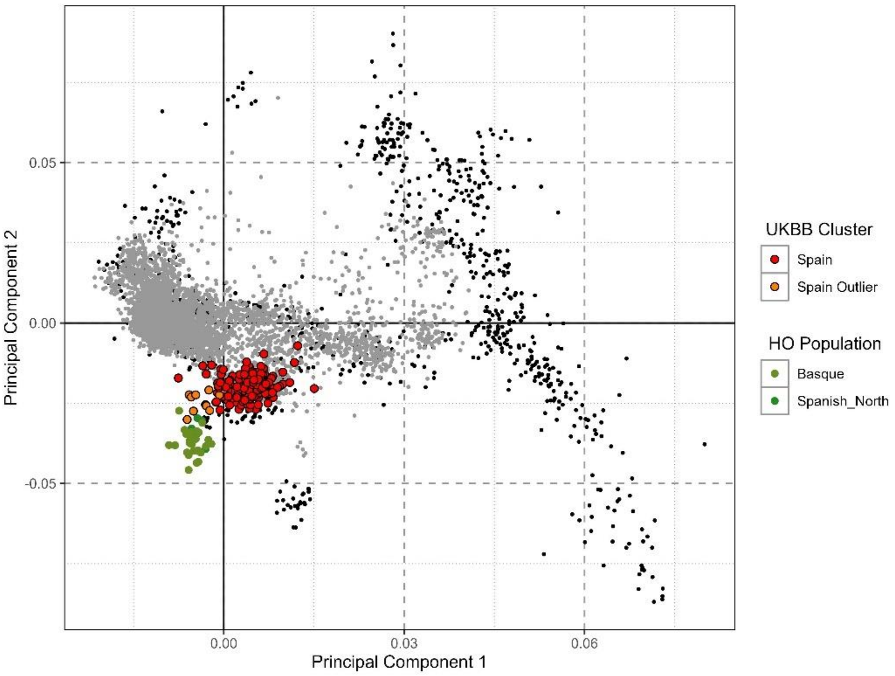

Given the genetic distinctiveness of these outliers, their elevated haplotype sharing consistent with a degree of isolation relative to the other UK Biobank individuals with a Spanish place of birth, and their projection towards Basque genetic space in PCA, we infer these individuals to be of northern Spanish or Basque ancestry. Previous genetic analyse of the Iberian Peninsula[12] has shown this area of Spain to be genetically distinct and would be consistent with the profile presented by these twelve individuals sampled from the UK Biobank.

References

1. Bycroft, C. *et al.* The UK Biobank resource with deep phenotyping and genomic data. *Nature* **562**, 203-209 (2018).
2. Lazaridis, I. *et al.* Genomic insights into the origin of farming in the ancient Near East. *Nature* **536**, 419-24 (2016).
3. Traag, V.A., Waltman, L. & van Eck, N.J. From Louvain to Leiden: guaranteeing well-connected communities. *Sci Rep* **9**, 5233 (2019).
4. Purcell, S. *et al.* PLINK: a tool set for whole-genome association and population-based linkage analyses. *Am J Hum Genet* **81**, 559-75 (2007).
5. Chang, C.C. *et al.* Second-generation PLINK: rising to the challenge of larger and richer datasets. *Gigascience* **4**, 7 (2015).
6. Alexander, D.H., Novembre, J. & Lange, K. Fast model-based estimation of ancestry in unrelated individuals. *Genome Res* **19**, 1655-64 (2009).
7. Lawson, D.J., Hellenthal, G., Myers, S. & Falush, D. Inference of population structure using dense haplotype data. *PLoS Genet* **8**, e1002453 (2012).
8. Leslie, S. *et al.* The fine-scale genetic structure of the British population. *Nature* **519**, 309-314 (2015).
9. Karakachoff, M. *et al.* Fine-scale human genetic structure in Western France. *Eur J Hum Genet* **23**, 831-6 (2015).
10. Raveane, A. *et al.* Population structure of modern-day Italians reveals patterns of ancient and archaic ancestries in Southern Europe. *Sci Adv* **5**, eaaw3492 (2019).
11. Pankratov, V. *et al.* Differences in local population history at the finest level: the case of the Estonian population. *Eur J Hum Genet* **28**, 1580-1591 (2020).
12. Bycroft, C. *et al.* Patterns of genetic differentiation and the footprints of historical migrations in the Iberian Peninsula. *Nat Commun* **10**, 551 (2019).
13. Patterson, N., Price, A.L. & Reich, D. Population structure and eigenanalysis. *PLoS Genet* **2**, e190 (2006).
14. Durbin, R. Efficient haplotype matching and storage using the positional Burrows-Wheeler transform (PBWT). *Bioinformatics* **30**, 1266-72 (2014).
15. Nelson, M.R. *et al.* The Population Reference Sample, POPRES: a resource for population, disease, and pharmacological genetics research. *Am J Hum Genet* **83**, 347-58 (2008).
16. Novembre, J. *et al.* Genes mirror geography within Europe. *Nature* **456**, 98-101 (2008).
17. Manichaikul, A. *et al.* Robust relationship inference in genome-wide association studies. *Bioinformatics* **26**, 2867-73 (2010).
18. Delaneau, O., Zagury, J.F., Robinson, M.R., Marchini, J.L. & Dermitzakis, E.T. Accurate, scalable and integrative haplotype estimation. *Nat Commun* **10**, 5436 (2019).
19. Danecek, P. *et al.* Twelve years of SAMtools and BCFtools. *Gigascience* **10**(2021).
20. Lazaridis, I. *et al.* Ancient human genomes suggest three ancestral populations for present-day Europeans. *Nature* **513**, 409-13 (2014).
21. Haak, W. *et al.* Massive migration from the steppe was a source for Indo-European languages in Europe. *Nature* **522**, 207-11 (2015).
22. Byrne, R.P. *et al.* Insular Celtic population structure and genomic footprints of migration. *PLoS Genet* **14**, e1007152 (2018).
23. van der Maaten, L.J.P. & Hinton, G.E. Visualizing high-dimensional data using t-SNE. *J. Mach. Learn. Res* **9**, 2579–605 (2008).
24. Gilbert, E. *et al.* The genetic landscape of Scotland and the Isles. *Proc Natl Acad Sci U S A* **116**, 19064-19070 (2019).
25. Ebenesersdottir, S.S. *et al.* Ancient genomes from Iceland reveal the making of a human population. *Science* **360**, 1028-1032 (2018).
26. Leblond, C.S. *et al.* Both rare and common genetic variants contribute to autism in the Faroe Islands. *NPJ Genom Med* **4**, 1 (2019).

27. Khrunin, A.V. *et al.* A genome-wide analysis of populations from European Russia reveals a new pole of genetic diversity in northern Europe. *PLoS One* **8**, e58552 (2013).
28. Naseri, A. *et al.* Personalized genealogical history of UK individuals inferred from biobank-scale IBD segments. *BMC Biol* **19**, 32 (2021).
29. Xue, J., Lencz, T., Darvasi, A., Pe'er, I. & Carmi, S. The time and place of European admixture in Ashkenazi Jewish history. *PLoS Genet* **13**, e1006644 (2017).
30. Browning, S.R. & Browning, B.L. Accurate Non-parametric Estimation of Recent Effective Population Size from Segments of Identity by Descent. *Am J Hum Genet* **97**, 404-18 (2015).
